# Low-pass whole genome sequencing of circulating tumor cells to evaluate chromosomal instability in triple-negative breast cancer

**DOI:** 10.1038/s41598-024-71378-3

**Published:** 2024-09-03

**Authors:** Serena Di Cosimo, Marco Silvestri, Cinzia De Marco, Alessia Calzoni, Maria Carmen De Santis, Maria Grazia Carnevale, Carolina Reduzzi, Massimo Cristofanilli, Vera Cappelletti

**Affiliations:** 1 Department of Advanced Diagnostics, Fondazione IRCCS Istituto Nazionale Dei Tumori Di Milano, Via Venezian 1, 20100 Milan, Italy; 2 Isinnova S.R.L, Brescia, Italy; 3 Department of Information Engineering, University of Brescia, Brescia, Italy; 4 Department of Radiation Oncology, Fondazione IRCCS Istituto Nazionale Dei Tumori Di Milano, Milan, Italy; 5 Breast Unit, Fondazione IRCCS Istituto Nazionale Dei Tumori Di Milano, Milan, Italy; 6 Division of Hematology-Oncology, Weill Cornell Medicine, New York, NY USA

**Keywords:** Chromosomal instability, Large-scale transitions, Circulating tumor cells, Triple-negative breast cancer, Copy number alterations, Breast cancer, Cancer genomics, Tumour biomarkers

## Abstract

Chromosomal Instability (CIN) is a common and evolving feature in breast cancer. Large-scale Transitions (LSTs), defined as chromosomal breakages leading to gains or losses of at least 10 Mb, have recently emerged as a metric of CIN due to their standardized definition across platforms. Herein, we report the feasibility of using low-pass Whole Genome Sequencing to assess LSTs, copy number alterations (CNAs) and their relationship in individual circulating tumor cells (CTCs) of triple-negative breast cancer (TNBC) patients. Initial assessment of LSTs in breast cancer cell lines consistently showed wide-ranging values (median 22, range 4–33, mean 21), indicating heterogeneous CIN. Subsequent analysis of CTCs revealed LST values (median 3, range 0–18, mean 5), particularly low during treatment, suggesting temporal changes in CIN levels. CNAs averaged 30 (range 5–49), with loss being predominant. As expected, CTCs with higher LSTs values exhibited increased CNAs. A CNA-based classifier of individual patient-derived CTCs, developed using machine learning, identified genes associated with both DNA proliferation and repair, such as *RB1*, *MYC*, and *EXO1*, as significant predictors of CIN. The model demonstrated a high predictive accuracy with an Area Under the Curve (AUC) of 0.89. Overall, these findings suggest that sequencing CTCs holds the potential to facilitate CIN evaluation and provide insights into its dynamic nature over time, with potential implications for monitoring TNBC progression through iterative assessments.

## Introduction

Breast cancer is a global health issue with approximately two and a half million new cases diagnosed annually worldwide^1^. Despite advances in screening, detection, and treatment, breast cancer remains the leading cause of cancer-related deaths among women^1^. The triple-negative (TNBC) subtype has the worst prognosis, emphasizing the need for improved care for both localized and metastatic patients^2^.

Chromosomal Instability (CIN) refers to the increased acquisition or loss of whole or fragmented chromosomes, and represents the most common form of genome instability in breast cancer^3^. Thus, improving our ability to assess CIN could offer promising insights into tumor progression and optimize patient care. Standard methods for evaluating CIN, such as DNA image cytometry and fluorescence in situ hybridization (FISH), are seldom used in the clinics due to their labor-intensive procedures and lack of high-throughput capabilities^4^. Alternative approaches including CIN70^5^ and HET70^6^ signatures, based on the expression of genes associated with aneuploidy and karyotype heterogeneity, or comparative genomic hybridization^7^ have also been utilized, showing that increased CIN is associated with metastatic potential and dismal prognosis^5–7^. However, bulk analytical methods give a broad view of CIN without distinguishing between ongoing or past events that may not have continued. In addition, DNA image cytometry, FISH, and transcriptomic analysis face challenges in capturing the inherent cell-to-cell heterogeneity of CIN as they rely on pooled DNA samples^4^.

Single-cell sequencing (scDNAseq) is emerging as a promising approach to tackle the above listed challenges by providing accurate and quantitative CIN measures that are amenable to clinical use^8^. scDNAseq can provide insights into the underlying aberrant molecular pathways driving CIN, with DNA repair genes being prominent candidates^8^. Additionally, scDNAseq overcomes limitations and confounding factors associated with the use of bulk tissue, such as surrounding stromal tissue, tumor heterogeneity, and limited sample availability^8^. Importantly, scDNAseq can be applied to circulating tumor cells (CTCs), which are emerging as a significant resource for timely breast cancer molecular characterization^9^. Unlike invasive tumor tissue biopsy that is prone to sampling error, CTCs allow dynamic and repeatable assessment, representing the ideal source for longitudinal measuring of an evolving feature such as CIN^10^.

In this study, we leveraged our expertise in CTC genotyping by next-generation sequencing^11^ to analyze CIN and underlying molecular alterations in TNBC patients. Specifically, we challenged low-pass Whole Genome Sequencing (lp-WGS) to determine the number of Large-Scale Transitions (LSTs) defined as contiguous regions of chromosomal breakage spanning at least 10 Mb^12^. The LST metric was chosen for its frequent use as a biomarker of CIN^8,13^. First, we tested the consistency of LST measurements using lp-WGS in a panel of breast cancer cell lines. Next, we extended our analyses to individual patient-derived CTCs collected at different clinical time-points, i.e., baseline, treatment, follow-up, and relapse. Finally, we developed a streamlined model for assessing CIN based on CTC copy number alterations (CNAs) within a specific set of genes.

## Results

**Fig. 1:**
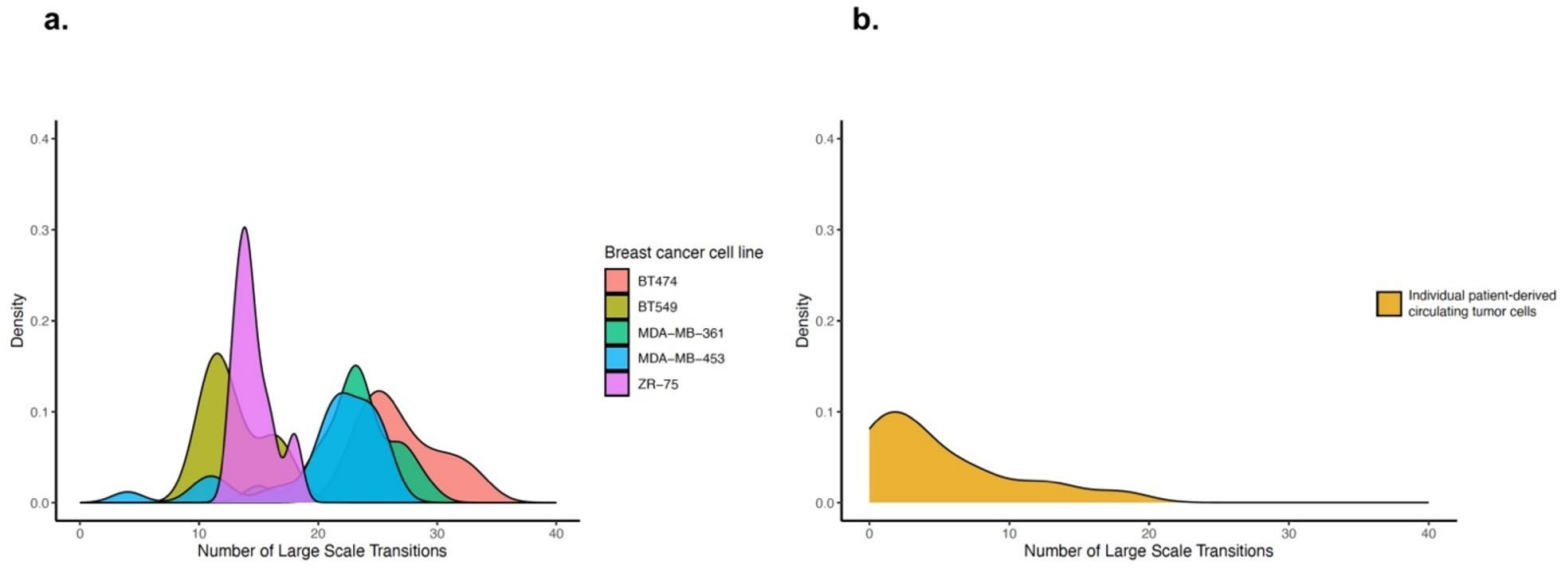
Large-scale transitions in breast cancer cell lines and patient-derived individual CTCs. Distribution of large- scale transitions (LSTs)—defined as chromosomal breakpoints between adjacent regions spanning at least 10 megabases—in breast cancer cell lines (**a**) and patient-derived individual CTCs (**b**).

As part of technical feasibility, we initially evaluated LSTs as a means of CIN evaluation in breast cancer cell lines undergoing whole genome amplification and lp-WGS at the single-cell level. The analyses were conducted on MDA-MB-453, MDA-MB-361, BT474, BT549, and ZR-75 cell lines in replicates as reported in Table 1. We observed a wide range of LSTs (median 22, range 4–33), reflecting the heterogeneous nature of CIN both within individual cells and across different cell lines (Fig. 1a).

**Table 1:** Reproducibility of LST values determined by lp-WGS in breast cancer cell lines.

Cell line	LSTs			
	Mean	SD	p*	Coefficient of variation**
MDA-MB-453 (n = 26)	20.4	5.5	0.9	0.27
BT474 (n = 9)	27.0	3.4	< 0.001	0.12
ZR-75 (n = 8)	14.6	1.7	< 0.001	0.11
BT549 (n = 7)	13.0	2.5	< 0.001	0.19
MDA-MB-361 (n = 18)	23.2	3.3	0.01	0.14

*p value as determined using Student's t-test.

**Coefficient of variation defined as the ratio of the standard deviation to the mean.

Large scale transitions, LSTs; standard deviation, SD; replicates, n.

Notably, LSTs values were significantly and reproducibly determined for the tested cell lines (Table 1).

We next analyzed clinical samples from 12 patients with histologically confirmed TNBC, successfully profiling (> 400,000 reads) a total of 35 CTCs collected at various time points throughout the disease trajectory (Table 2).

**Table 2:** Triple negative breast cancer patient and CTC characteristics.

Cohort size, age, and disease presentation	
Number of patients	12
Number of blood samples per patient, median (range)	1 (1–3)
Age at baseline, median (range), years	43 (39–75)
Germ-line *BRCA*	
Wild type	1
Mutated	4
Unknown	7
Stage of disease	
Early	4
Metastatic	5
No evidence of disease	3
CTC detection	
Overall number of collected CTCs	42
Number per patient, median (range)	3 (1–10)
Number of sequenced CTCs	35
Timing of collection of sequenced CTCs	
During treatment	13
Follow-up	4
At relapse	18

LSTs in CTCs showed heterogeneity (median 3, range 0–18), with values lower than those observed in cell lines, especially during treatment (median 2, range 0–13). Median LSTs in CTCs from patients with and without metastases were 2 and 3.5, respectively; 3 in germ-line *BRCA* mutation carriers*.* The distribution of LSTs values displayed a bimodal shape (Fig. 1b). However, its limited extent prevented definition of a clear threshold, prompting the use of the median number of LSTs to classify CTCs as either LST-low (number of LSTs < 3) or high (number of LSTs ≥ 3).

**Fig. 2:**
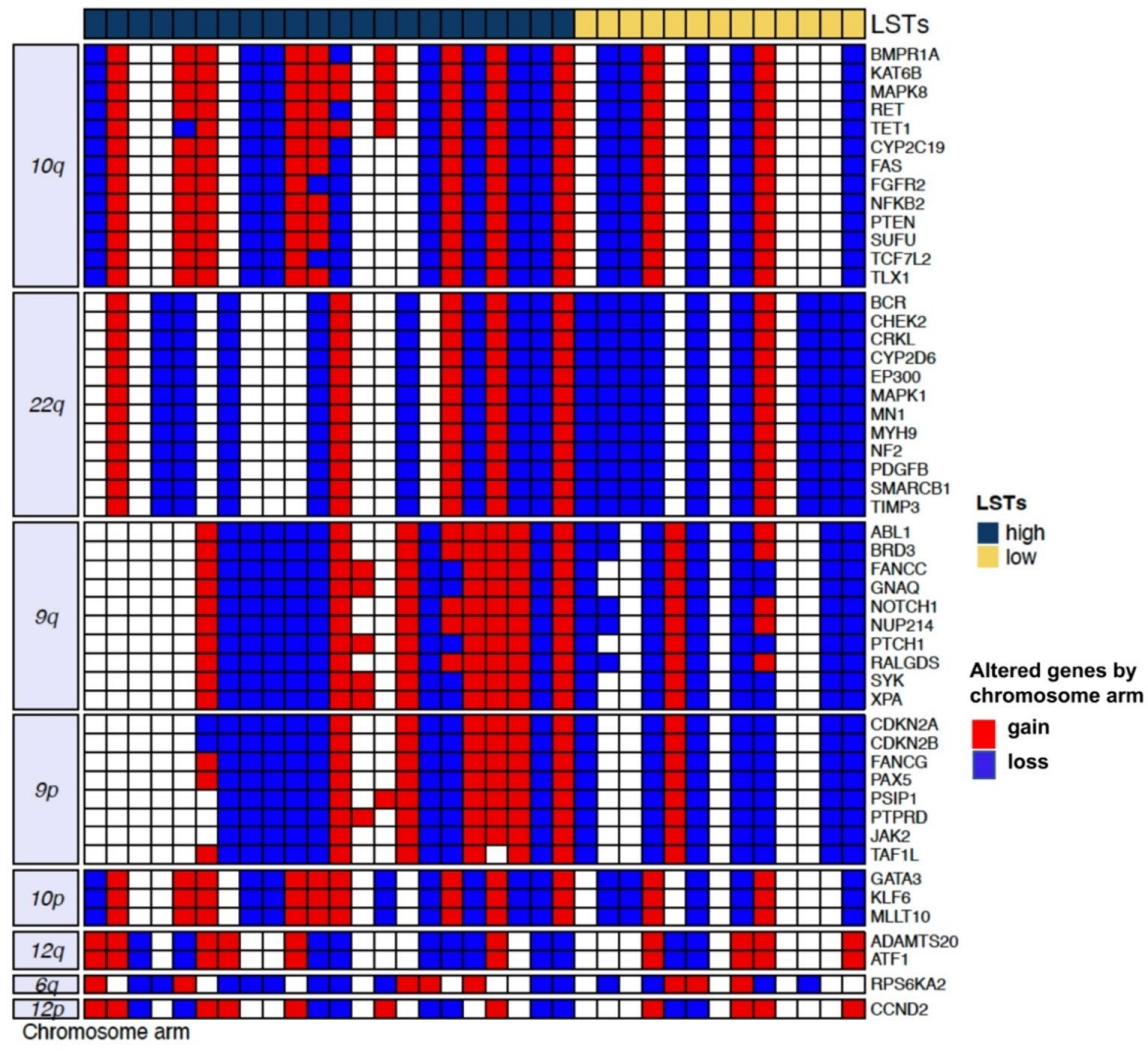
Copy number alterations in individual CTCs of TNBC patients. The heatmap shows CTCs in the columns according to their number of LSTs and classified as high when ≥ 3 (dark blue) or low when < 3 (yellow). The rows show the top-fifty altered genes by chromosomal arm, with red indicating gain and blue indicating loss.

We next analyzed the CTC CNA profile. The mean number of CNAs per CTC was 30 (range 5–49), with deletions outnumbering amplifications at 401:291 (Supplementary Fig. 3). The most frequently lost or gained chromosomal regions and the corresponding genes are reported in Fig. 2.

Recurrent alterations involved 9p and 9q, containing *ABL1*, *NOTCH1*, and *CDKN2A*; 10, containing *MAPK8* and *GATA3*; and 22q, containing *BCR*, as expected and consistently with literature on genes involved in TNBC oncogenesis^14^. We also analyzed CNA with respect to LSTs. Compared to CTCs classified as LST-low, those with higher values had a numerical increase in CNAs overall, median CNAs in CTCs with high and low LSTs 22 and 13, p = 0.08, and a prevalence of copy number losses, particularly in homologous recombination deficiency (HDR) related genes, with 59% (13/22) of CTCs classified as LST-high and 31% (4/13) of the LST-low showing *RAD51*, *BLM*, or *WNR* copy loss, p = 0.05. Oncogenic signaling pathways analysis showed that CTCs classified as LST-high were enriched for CNAs—either gains or losses—affecting NRF2, TP53, and TGF-beta signaling (Supplementary Fig. 1).

However, the question remained as to which factors most strongly influence LSTs. Therefore, we used a Random Forrest (RF) non parametric machine learning method to develop a CNA-based classifier of patient-derived CTCs with and without LSTs (Supplementary Fig. 2).

**Fig. 3:**
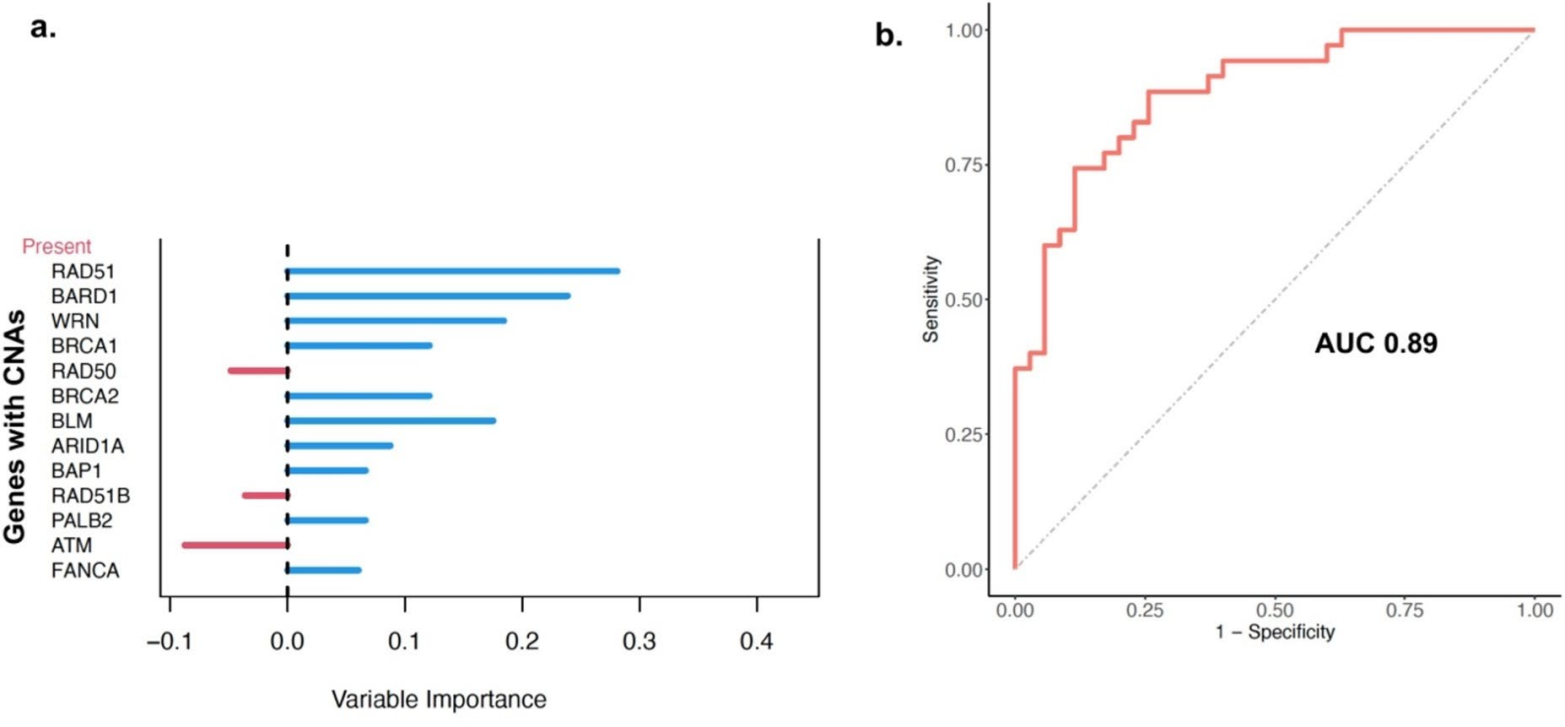
Model performance evaluation. (**a**) Internal measure of variable importance (VIMP) of altered genes in CTCs harboring CIN. The VIMP shows decreases in classification accuracy when the values of a given variable are randomly permuted, while all other predictors remain unchanged in the model. The larger the VIMP of a variable, the more predictive the variable (**b**) Receiver operating characteristic curve (ROC) for prediction of LSTs based on the CNAs of breast cancer related genes profiled by lp-WGS and computed through a RF learning model. AUC (Area under the curve).

A total of 39 covariates were included in the model, consisting of CNAs of established HDR related^15^ and TNBC driver^16^ genes (Supplementary Table 1). *RB1*, *MYC*, and *EXO1* emerged as the most relevant predictors of CIN among all covariates, with variable importance index (VIMP) indicating that the prediction error rate would increase by up to 30% if the CNAs of these genes were randomly permuted in the model (Fig. 3a).

Strikingly, the RF model yielded an AUC of 0.89 indicating that the analysis of CNAs in a few genes might be sufficient to achieve reliable classification of CIN (Fig. 3b).

## Discussion

Chromosomal instability is increasingly recognized as a cancer hallmark, crucial in initiation, progression, and metastasis, with implications for optimizing care^3,17^. However, its regular assessment is hindered by its dynamic nature and limitations in currently available tools^4^. Hence, there is a critical need to develop CIN biomarkers that are easily and reliably assessable to inform and guide clinical management, including in breast cancer patients. To the best of our knowledge, several studies have assessed the CNA of CTCs, but none have tackled CIN analysis ^18–20^. In this study, we analyzed lp-WGS data to evaluate LSTs and CNAs in individual CTCs from women with TNBC, and to build a predictive classifier of CIN at the single-cell level achieving an AUC of 0.89. While our study is preliminary, we are the first to report a cost-effective sequencing assay such as lp-WGS for assessing LSTs in CTCs, the utilization of distinctive genetic features to evaluate complex phenomena, and ultimately, the development of a performing predictive model based on CNAs interactions. Additionally, we incorporated the assessment of CIN, a dynamic variable on CTCs, whose analysis can be repeated over time through a minimally invasive blood draw. These findings not only pave the way to a novel analytical approach for assessing CIN but also provide significant contributions to the field.

The distributions of LSTs values, both in breast cancer cell lines and individual CTCs, confirm the significant heterogeneity of CIN. This observation is consistent with existing literature, which suggests that the CIN underlying mechanisms leading to dysfunctional chromosome duplication and segregation can vary^21^. Interestingly, the LSTs values observed in CTCs, particularly those from recurrent patients, were not as elevated as expected. These findings align with prior research indicating low karyotypic variance during disease progression across various cancer types including the breast^22^. To reconcile this observation with the well-documented prevalence of CIN in cancer, the theory of the CIN paradox posits that tumors typically exhibit intermediate levels of CIN as excessively high levels are detrimental, while insufficient levels do not guarantee an advantage in terms of proliferation and survival^23^. In addition, the low LST values observed in recurrent breast cancer patients may be influenced by the number of CTCs analyzed potentially affecting the prevalence of CIN. This raises the question of deriving individuals' features from their single-cell data. To the best of our knowledge, few previous work estimated the required sample size, i.e., the number of cells to profile, to infer CIN from scDNAseq data^24^. Regarding CTCs, while some have suggested diagnosing cancer with CIN based on the presence of only one^25^ to at least 3 unstable CTCs^26^, it is uncertain if this also applies to breast cancer. Therefore, further research is needed.

Several studies have characterized CNAs in TNBC tissue using high-resolution genomic data^16^. Consistent with these findings, CTC CNAs more frequently showed deletions than amplifications. Despite potential limitations of lp-WGS compared to higher resolution next-generation sequencing, we report that CTC chromosomal gains and losses occurred in regions where breast cancer-related genes are generally found, supporting that our findings were unlikely to be due to random sequencing dropout or due to amplification bias. For instance, *CDKN2A* and *NOTCH1* were identified in loss regions^14,16^. It is also not surprising that CTCs with high LSTs were more frequently characterized by the loss of HDR related genes. However, whether this is the cause of LSTs or if, conversely, the loss of these genes is the consequence, we cannot ascertain. The fact remains that DNA repair genes alone do not fully explain CTC CIN. As already reported for tumor tissue, other factors such as mitotic errors, replication stress, telomere crisis, and breakage fusion bridge cycles^21^, among others, may also be at play. Therefore, we hypothesized that the simultaneous analysis of copy number changes in a set of selected genes could help define CTCs with and without LSTs. To this end, we utilized, for the first time in this context, the RF learning model which allowed us to examine the impact of different potential predictors in creating a predictive model^27^. Our findings indicate that *RB1*, *EXO1*, and *MYC* are the most significant predictors among all covariates for identifying LSTs, with a variable importance index exceeding 30%. These results align with preclinical evidence suggesting that the loss of G1/S control resulting from RB1 pathway inactivation, coupled with *MYC*-induced mitogen addition and DNA damage, leads to chromatid breaks and chromatid cohesion defects in mitotic cells^28^. These aberrations ultimately contribute to aneuploidy in the offspring cell population. Furthermore, LSTs represent a subset of chromosomal rearrangements, particularly evident when double-strand breaks are repaired through non-homologous end joining, as observed in BRCA-deficient environments^12^. Aligned with this, alterations of *BRCA1* and *BRCA2* demonstrated substantial predictive value within the developed classifier.

This study and its methods have several strengths, as the classifier presented here represents a resource for a deeper understanding of the origins and diversity of CIN. Our results focus attention on a narrow group of genes involved in fundamental cellular processes for maintaining genomic integrity. Additionally, our results support the broader application of CIN measures in clinical diagnostics, as sequencing techniques, which have been rarely used due to technical difficulties, are becoming more widespread and affordable every day. Finally, this work focuses on targets that may lead to potentially applicable therapies, beyond those traditionally suggested based on platinum^21^ and taxane^29^ for the most unstable tumors.

Despite these strengths, this study and the methods used also have weakness that should be noted. First, the number of LSTs is only one functional measure of CIN, and other measures exist, including telomere allele imbalance and loss of heterozygosis. Second, data on the single-cell nature of copy number or LST burden in single tumor cells in a large cohort are lacking, and technical limitations require that the data generated to date be interpreted with caution. Finally, RF cannot produce hypothesis testing results, such as relative risks, odds ratios, or p-values, as in classical regression methods, and its use is for model exploration. Hence, the data presented herein merit confirmation.

In conclusion, our study demonstrates the feasibility of low-resolution lp-WGS for assessing both LSTs and CNAs in TNBC CTCs at a single-cell level. As a proof-of-concept study, we developed a classifier of LSTs based on CNAs of genes involved both in HDR and replication process. Future research with larger sample sizes will be necessary to evaluate the clinical application of this assay, which lays the groundwork for leveraging CIN in precision oncology efforts.

## Materials and methods

### Sample processing

For spiking experiments, five cell lines broadly representative of breast cancer, expressing (+) or lacking (−) the estrogen receptor (ER), and showing Human Epidermal Growth Factor Receptor 2 amplified (HER2+) or normal (HER2−) status were purchased from the American Type Culture Collection (ATCC, Manassas, VA, USA). ZR75-1 (ER+/HER2−), MDA-MB-453 (ER−/HER2+), MDA-MB-361 (ER+/HER2+), and BT-549 (ER−/HER2−) were cultured in DMEM/F-12 (Lonza, Swizerland) medium supplemented with 10% fetal bovine serum, BT474 (ER+/HER2+) in Dulbecco’s Modified Eagle’s Medium (DMEM) (Sigma, Darmstadt, Germany). All culture media were supplemented with antibiotic–antimycotic Solution (100 ×) (Sigma, Darmstadt, Germany), 10% fetal bovine serum (FBS) (Sigma, Darmstadt, Germany) and L-glutamine (2 mM) (Invitrogen GmbH, USA), and tested negative for mycoplasma contamination. Single cells were manually captured under an inverted microscope using a p10 micropipette and directly spiked into healthy donor blood. Spiked-in samples were processed following the same protocols used for clinical samples.

Peripheral blood was collected from study patients in K2EDTA tubes (10 ml) and processed within 1 h of draw using the Parsortix platform (Angle plc, Guildford, UK) for size-based enrichment. Following enrichment, cells were harvested according to manufacturer’s instructions and fixed with 2% paraformaldehyde for 20 min at room temperature.

### Cell isolation, amplification and sequencing

Enriched patient samples were processed using the DEPArray system (Menarini Silicon Biosystems, Bologna, IT)^11^. Individual cells were sorted based on morphological characteristics, DNA content, and fluorescence labeling against epithelial (CK, EpCAM, EGFR) and leukocyte (CD45, CD14, CD16) markers, as previously reported^11^. Subsequently, white blood cells expressing only leukocyte markers and single CTCs expressing either only epithelial markers or lacking any marker were recovered for downstream molecular analyses. WGA was performed on single cells using the Ampli1™ WGA kit version 02 (Menarini Silicon Biosystems, Bologna, IT) as per manufacturer instructions. For single cells derived from blood (CTCs and WBC), the quality of the WGA product was determined using the Ampli1™ QC Kit (Menarini Silicon Biosystems, Bologna, IT). A genomic integrity index (GII) was allocated for each sample scored from 0 to 4. Only single cells with sufficiently good quality DNA as determined by a GII ≥ 2 were selected for downstream analysis.

### Low-pass whole genome sequencing and bioinformatics

Ampli1™ low-pass kit for Illumina (Menarini Silicon Biosystems, Bologna, IT) was used for preparing low-pass Whole Genome Sequencing (lpWGS) libraries from single cells. Forhigh-throughput processing, the manufacturer procedure was implemented in a fully automated workflow on Ion Torrent Ion S5-system (ThermoFisher, Waltham, MA, USA). Ampli1™ low-pass libraries were normalized and sequenced by Ion530 chip. The obtained FASTQ files were quality checked and aligned to the hg19 human reference sequence using tmap aligner tool on Torrent_Suite 5.10.0. and alignment (BAM) files were generated. All samples with < 400.000 reads were excluded from the analyses.

BAM files underwent quality filtering using qualimap^30^ and were processed using two separate pipelines for CIN and CNAs. Each chromosomal break between contiguous regions of at least 10 Mb was tabulated to calculate the number of large-scale transitions (LSTs) per CTC genome. Copy number alterations were identified using QDNAseq software (version 11.0) according to the following settings: minMapq = 37, window = 500 kb. “Gain” and “loss” calls were filtered out by residual (> 4 standard deviation, SD) and black list regions reported in ENCODE database. Segmented copy number data of each sample were extracted starting from log2Ratio value. For the purpose of CNA profile, chromosome 19 was not considered due to its biased deletion associated with the high CG base percentage. Samples were classified as aberrant if they exhibited either ≥ 1 genomic regions with amplification/deletion greater than 12.5 Mb, or if the cumulative amplification/deletion of different genomic regions exceeded 37.5 Mb. OncoKb database was interrogated to evaluate biological and clinical relevant CNAs in CTCs (access date: March 2024).

### Statistics

Biological analyses relied on canonical oncogenic signaling pathways, as previously defined^31^ and processed using custom functions from the maftools R package^32^, alongside Gene Ontology (GO) biological process terms and KEGG pathways via the ClusterProfiler Bioconductor package. CIN predictor was developed using the SMOTE method^33^ to address sample imbalance between presence and absence of LSTs. Classification was performed using the random forest algorithm on 39 genes^34^ with bootstrap re-sampling used to estimate standard errors and confidence intervals. The discriminatory capability of the CIN classifier was assessed using ROC curves and expressed by AUC values. Analyses of association were conducted using t-test for continuous variables, and Fisher test for categorical variables. All analyses were performed using R software (www.R-project.org), statistical significance was set at a p-value < 0.05.

### Conference presentation

These results have been presented in part at the Molecular Analysis for Precision Oncology (MAP) Congress, Amsterdam, Netherlands, Oct 14–16, 2022.

## Supplementary Information

Supplementary Information 1.

Supplementary Information 2.

Supplementary Information 3.

Supplementary Information 4.

Supplementary Information 5.

## Data Availability

Raw sequencing data are available from the corresponding author upon request.
