# Supplementary material for: Low-pass whole genome sequencing of circulating tumor cells to evaluate chromosomal instability in triple-negative breast cancer
Source: Sci Rep. 2024 Sep 3;14:20479. doi: 10.1038/s41598-024-71378-3 (PMC11372142; doi:10.1038/s41598-024-71378-3)
Supplement: Supplementary file 1 — Supplementary Information 1. [file 41598_2024_71378_MOESM1_ESM.docx]

**Supplementary Table 1** List of genes analyzed for the predictive model of chromosomal instability

| *ATM* |
| --- |
| *BRCA1* |
| *BRCA2* |
| *BRDT* |
| *CCND1* |
| *CCND3* |
| *CDH1* |
| *CDK6* |
| *CDKN2A* |
| *CDKN2B* |
| *DNMT3A* |
| *EGFR* |
| *ERBB2* |
| *ESR1* |
| *EXO1* |
| *FGFR1* |
| *FGFR2* |
| *IGF1R* |
| *KRAS* |
| *LIG4* |
| *MAP2K4* |
| *MAP3K1* |
| *MDM2* |
| *MSH2* |
| *MYC* |
| *NCOR1* |
| *NF1* |
| *PALB2* |
| *PDGFRA* |
| *PIK3CA* |
| *PTEN* |
| *RAD51B* |
| *RAD51C* |
| *RAD51D* |
| *RB1* |
| *SMAD4* |
| *TP53* |
| *ZNF217* |
| *ZNF703* |

**Supplementary Fig. 1**

The graphs show the fraction of CTCs affected by gain (a) or loss (b) in genes involved in ten signaling pathways according to high (blue bar) or low LSTs (yellow bar).

**Supplementary Fig. 2**

Out of bag prediction errors across number of trees used in random forest for all observation (black), absence (green, LSTs= 0) and presence (red, LSTs ≥ 1) of CIN.

**Supplementary Fig. 3**

**CNA profiles of the 35 sequenced CTCs. Blue and red colors refer to deletions and amplifications call. Log2 ratio along chromosomal location are reported on y and x axis, respectively. Standard deviation and expected standard deviation are reported on the top right of the plots.**
