# Supplementary figures and images for: Low-pass whole genome sequencing of circulating tumor cells to evaluate chromosomal instability in triple-negative breast cancer
Source: Sci Rep. 2024 Sep 3;14:20479. doi: 10.1038/s41598-024-71378-3 (PMC11372142; doi:10.1038/s41598-024-71378-3)

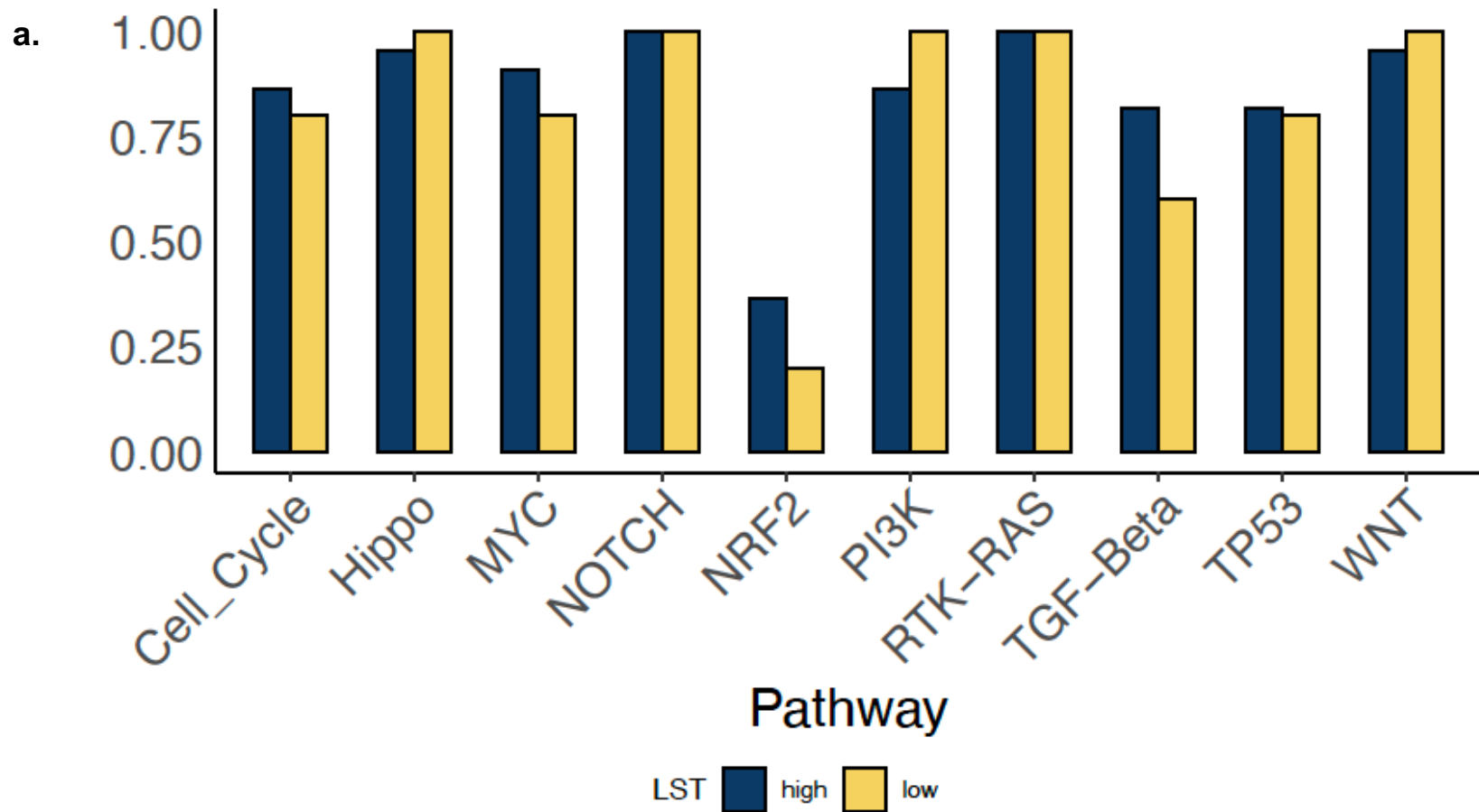

Supplement: Supplementary file 2 — Supplementary Information 2. [file 41598_2024_71378_MOESM2_ESM.pdf]

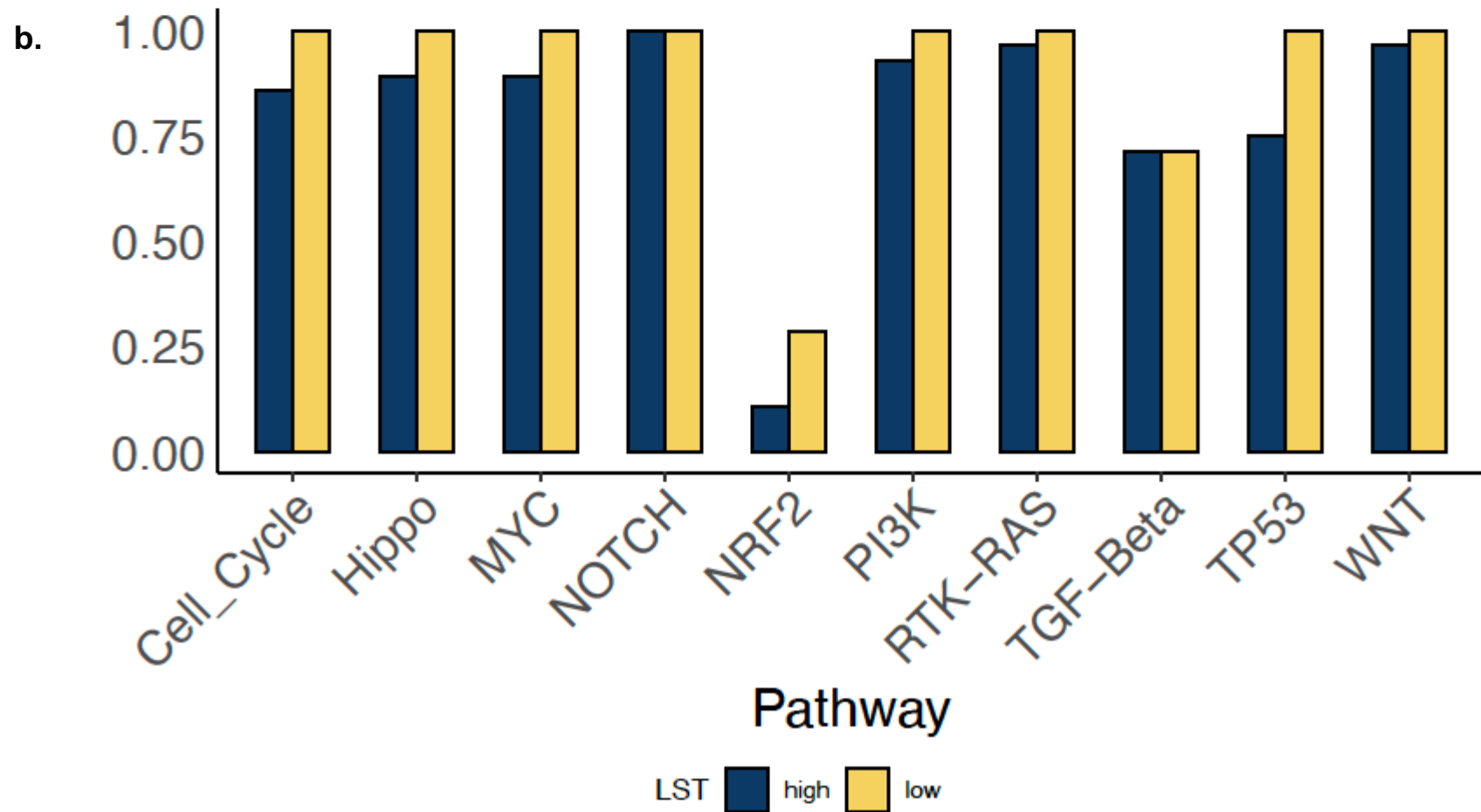

Supplement: Supplementary file 3 — Supplementary Information 3. [file 41598_2024_71378_MOESM3_ESM.pdf]

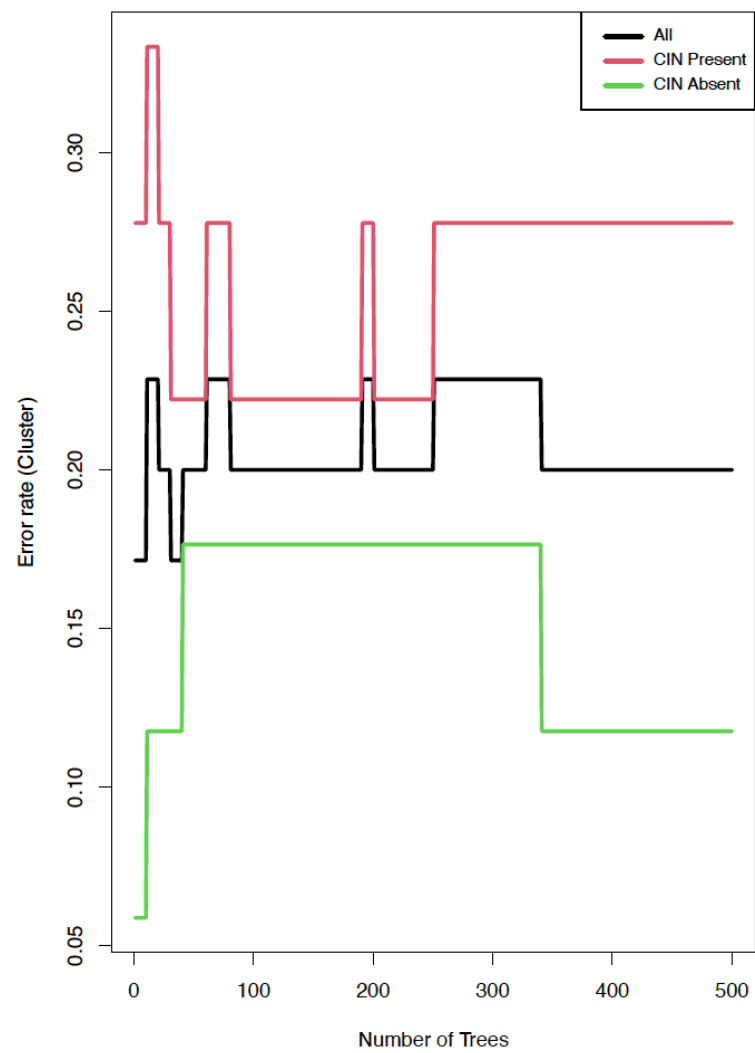

Supplement: Supplementary file 4 — Supplementary Information 4. [file 41598_2024_71378_MOESM4_ESM.pdf]
