# Supplementary material for: Low-pass whole genome sequencing of circulating tumor cells to evaluate chromosomal instability in triple-negative breast cancer
Source: Sci Rep. 2024 Sep 3;14:20479. doi: 10.1038/s41598-024-71378-3 (PMC11372142; doi:10.1038/s41598-024-71378-3)
Supplement: Supplementary file 5 — Supplementary Information 5. [file 41598_2024_71378_MOESM5_ESM.pdf]

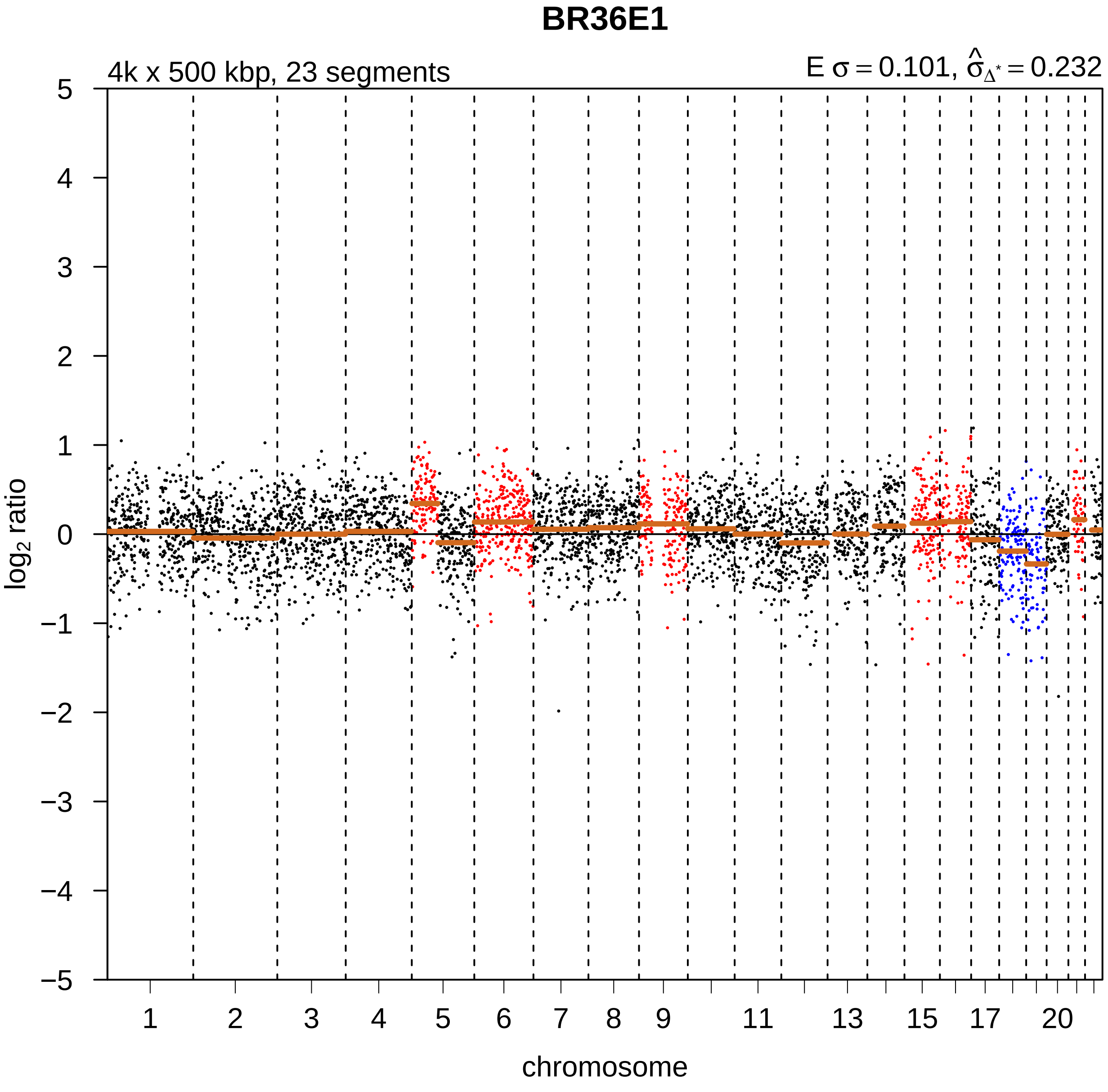

BR36E1
4k x 500 kbp, 23 segments
E σ = 0.101, σ̂Δ* = 0.232
5
4
3
2
1
0
−1
−2
−3
−4
−5
log2 ratio
1
2
3
4
5
6
7
8
9
11
13
15
17
20
chromosome

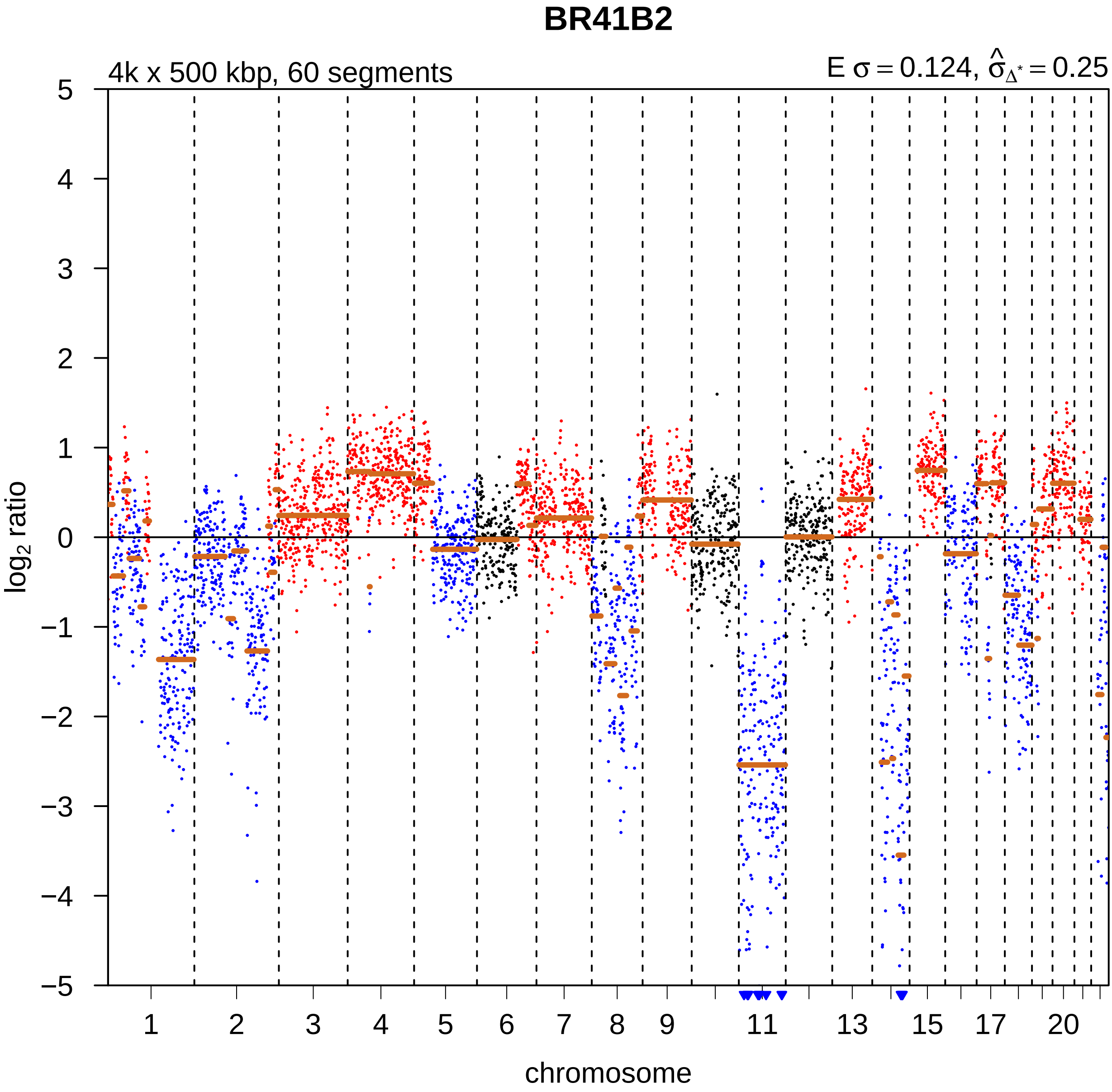

BR41B2
4k x 500 kbp, 60 segments
E σ = 0.124, σ̂Δ* = 0.25
log2 ratio
5
4
3
2
1
0
−1
−2
−3
−4
−5
1
2
3
4
5
6
7
8
9
11
13
15
17
20
chromosome

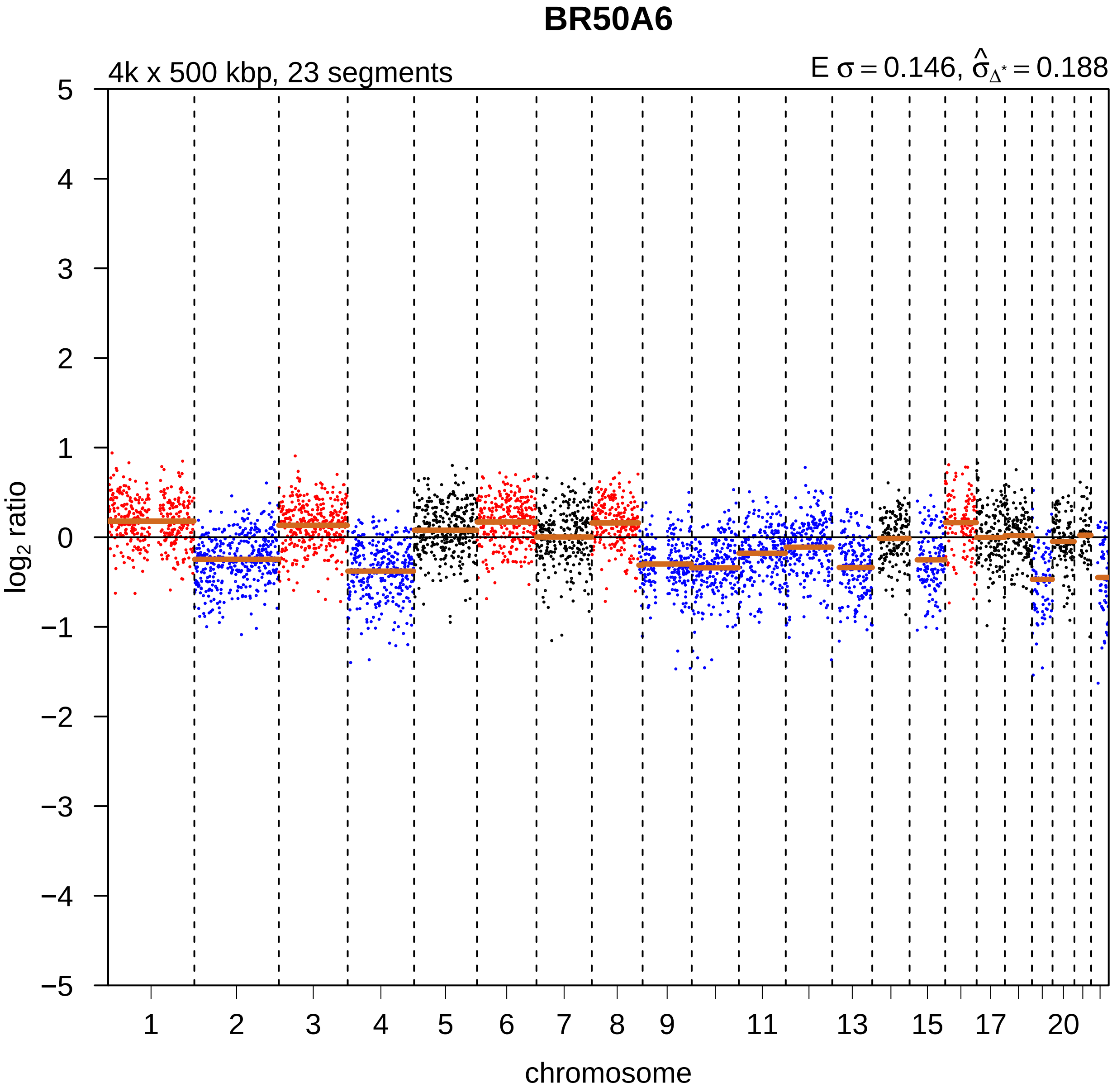

BR50A6
4k x 500 kbp, 23 segments
E σ = 0.146, $\hat{\sigma}_{\Delta^*}$ = 0.188
log2 ratio
5
4
3
2
1
0
−1
−2
−3
−4
−5
1
2
3
4
5
6
7
8
9
11
13
15
17
20
chromosome

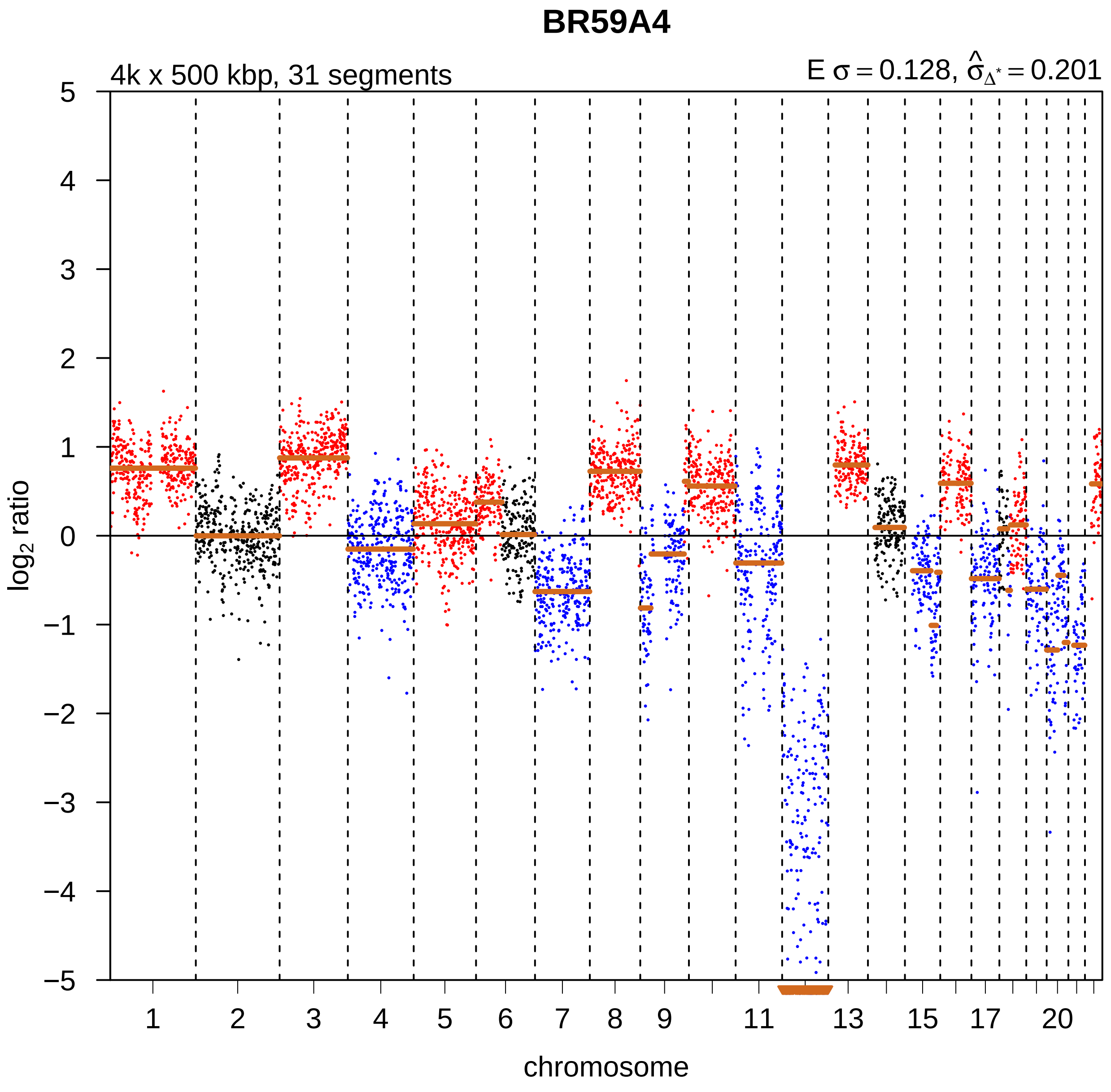

BR59A4
4k x 500 kbp, 31 segments
E σ = 0.128, $\hat{\sigma}_{\Delta^*}$ = 0.201
5
4
3
2
1
0
−1
−2
−3
−4
−5
$\log_2$ ratio
1
2
3
4
5
6
7
8
9
11
13
15
17
20
chromosome

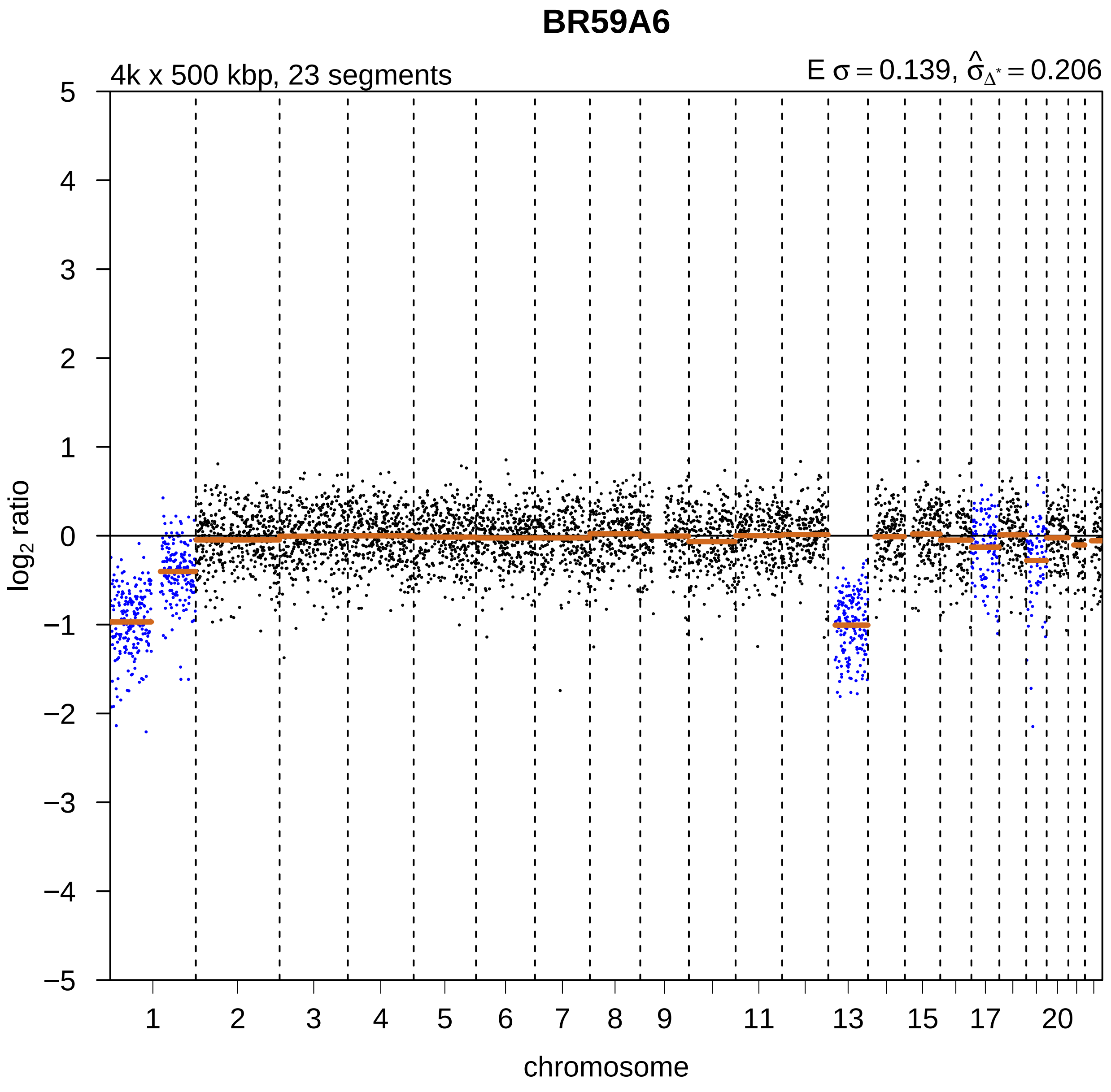
BR59A6
4k x 500 kbp, 23 segments
E σ = 0.139, $\hat{\sigma}_{\Delta^*}$ = 0.206
5
4
3
2
1
0
−1
−2
−3
−4
−5
log2 ratio
1
2
3
4
5
6
7
8
9
11
13
15
17
20
chromosome

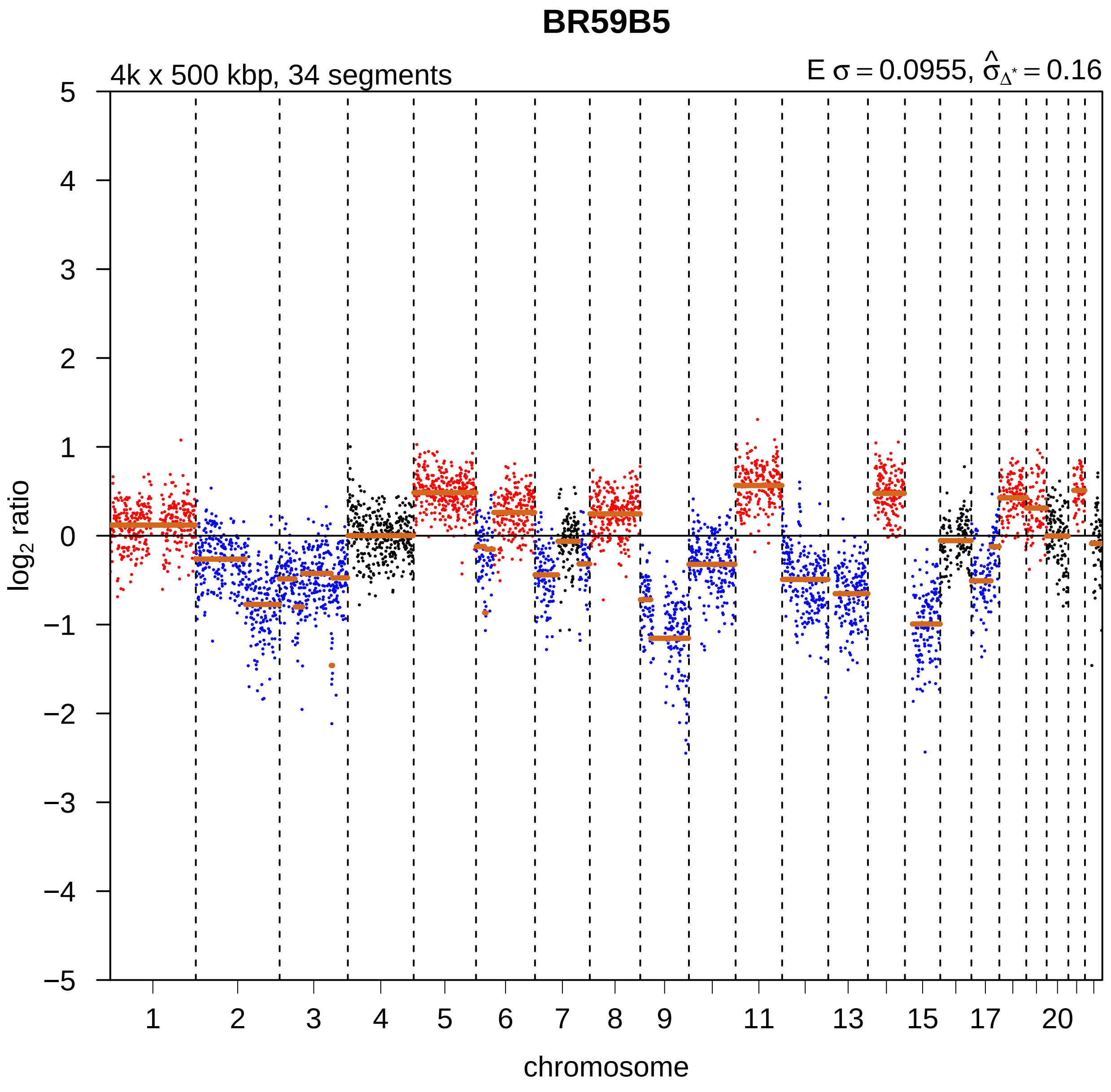
BR59B5
4k x 500 kbp, 34 segments
E σ = 0.0955, σ̂Δ* = 0.16
log2 ratio
5
4
3
2
1
0
−1
−2
−3
−4
−5
1
2
3
4
5
6
7
8
9
11
13
15
17
20
chromosome

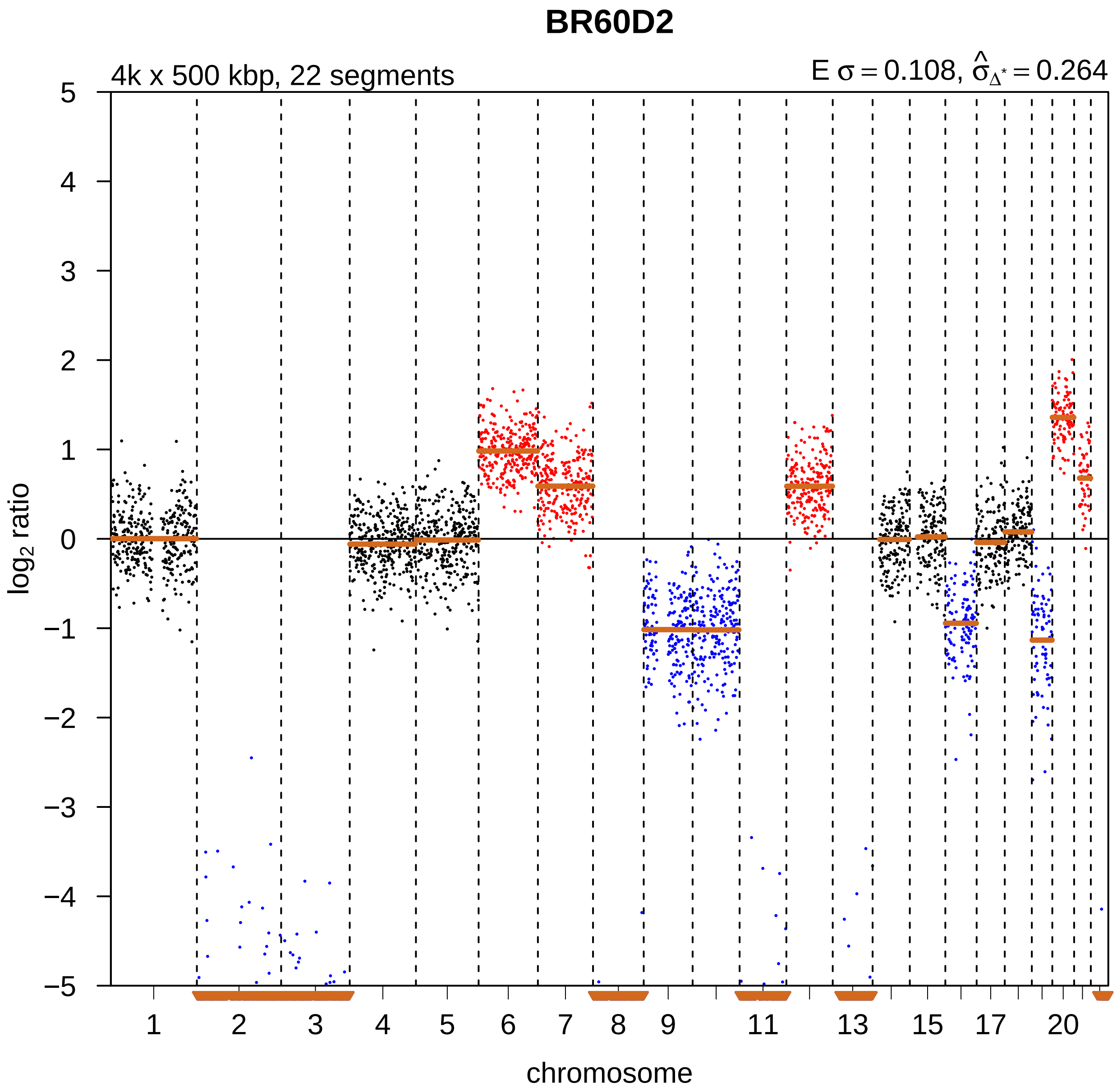

BR60D2
4k x 500 kbp, 22 segments
E σ = 0.108, $\hat{\sigma}_{\Delta^*}$ = 0.264
log2 ratio
5
4
3
2
1
0
−1
−2
−3
−4
−5
1
2
3
4
5
6
7
8
9
11
13
15
17
20
chromosome

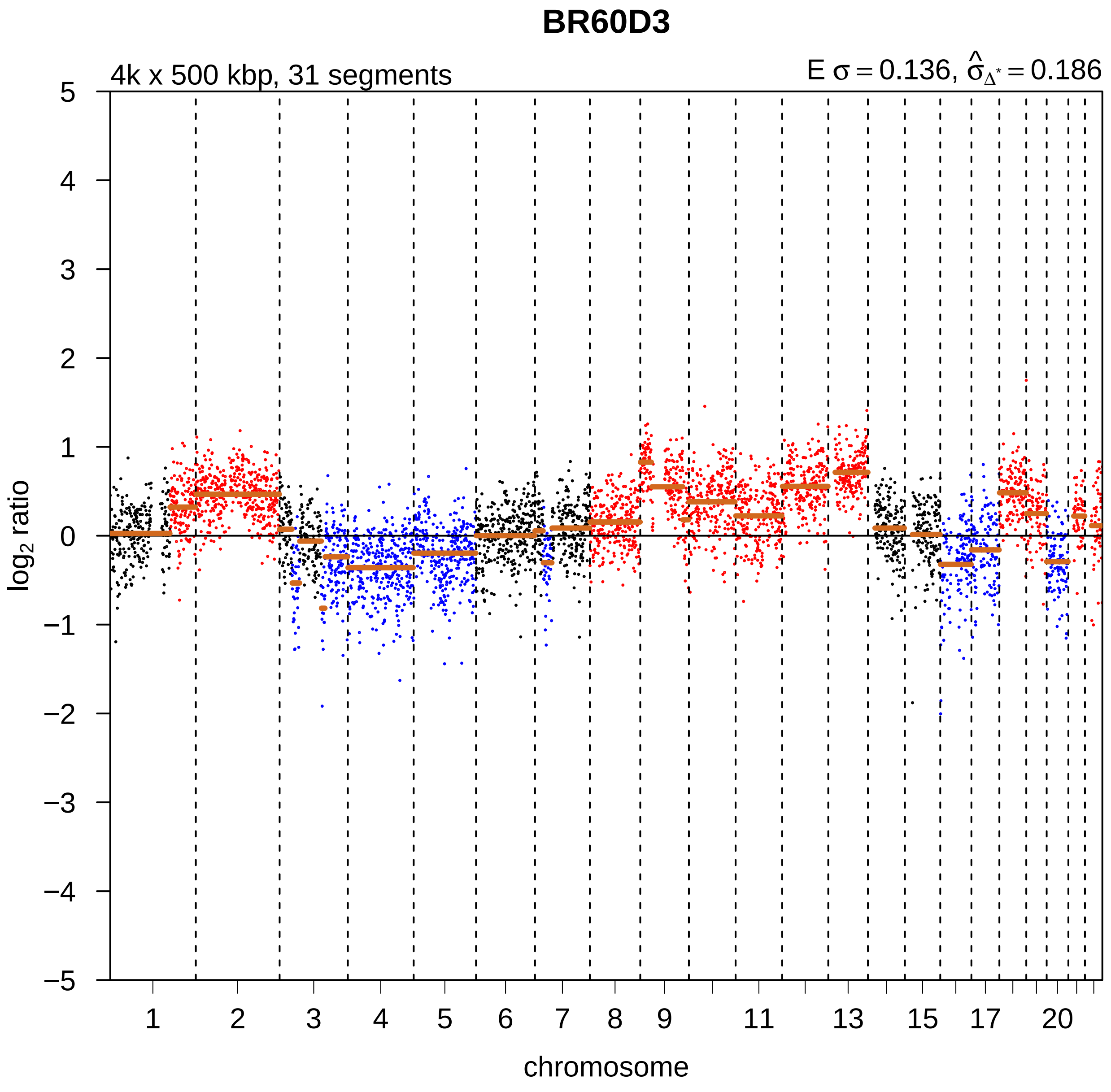
BR60D3
4k x 500 kbp, 31 segments
E σ = 0.136, $\hat{\sigma}_{\Delta^*}$ = 0.186
log2 ratio
5
4
3
2
1
0
−1
−2
−3
−4
−5
1
2
3
4
5
6
7
8
9
11
13
15
17
20
chromosome

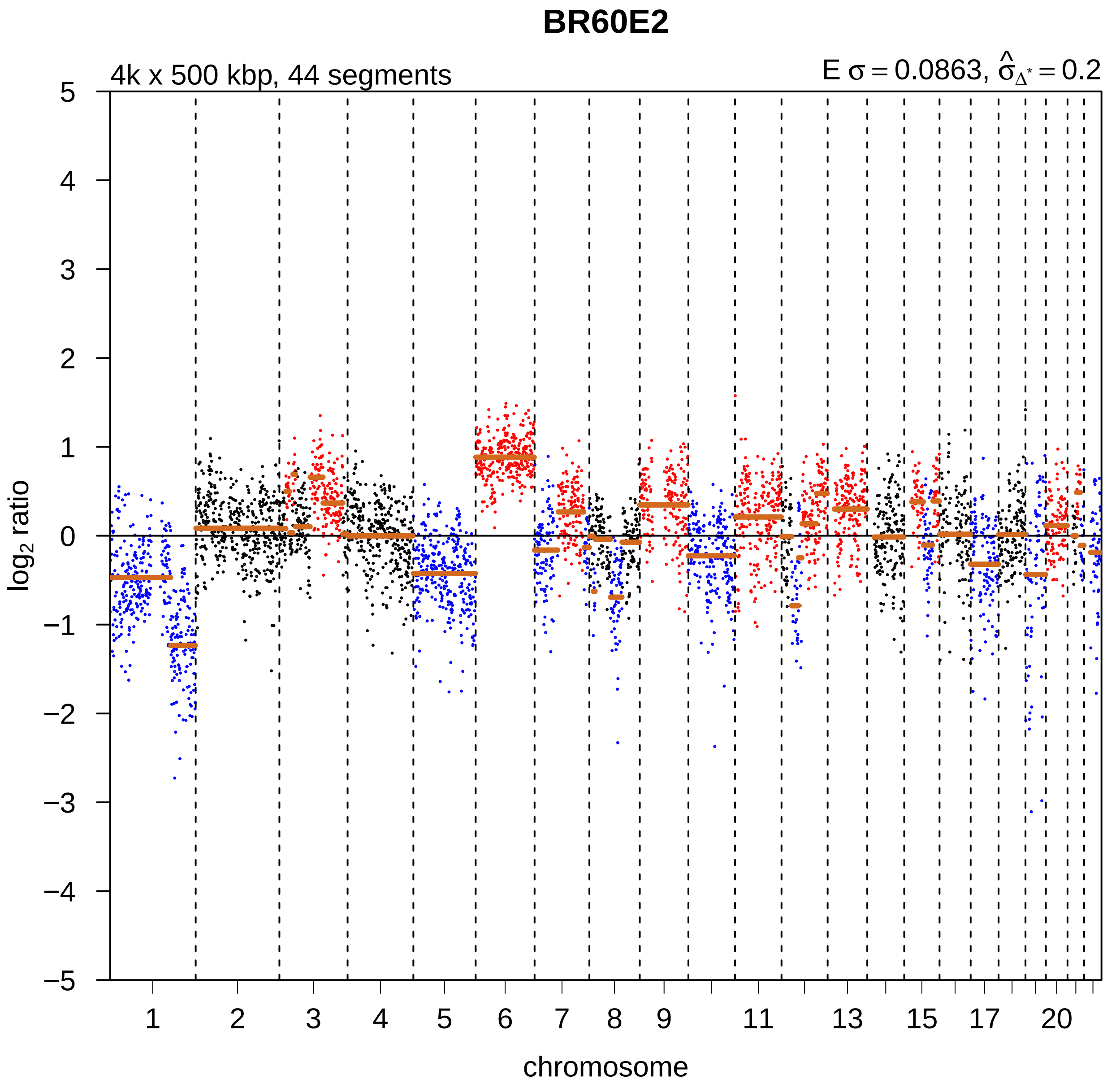

BR60E2
4k x 500 kbp, 44 segments
E σ = 0.0863, σ̂_Δ* = 0.2
log2 ratio
5
4
3
2
1
0
−1
−2
−3
−4
−5
1
2
3
4
5
6
7
8
9
11
13
15
17
20
chromosome

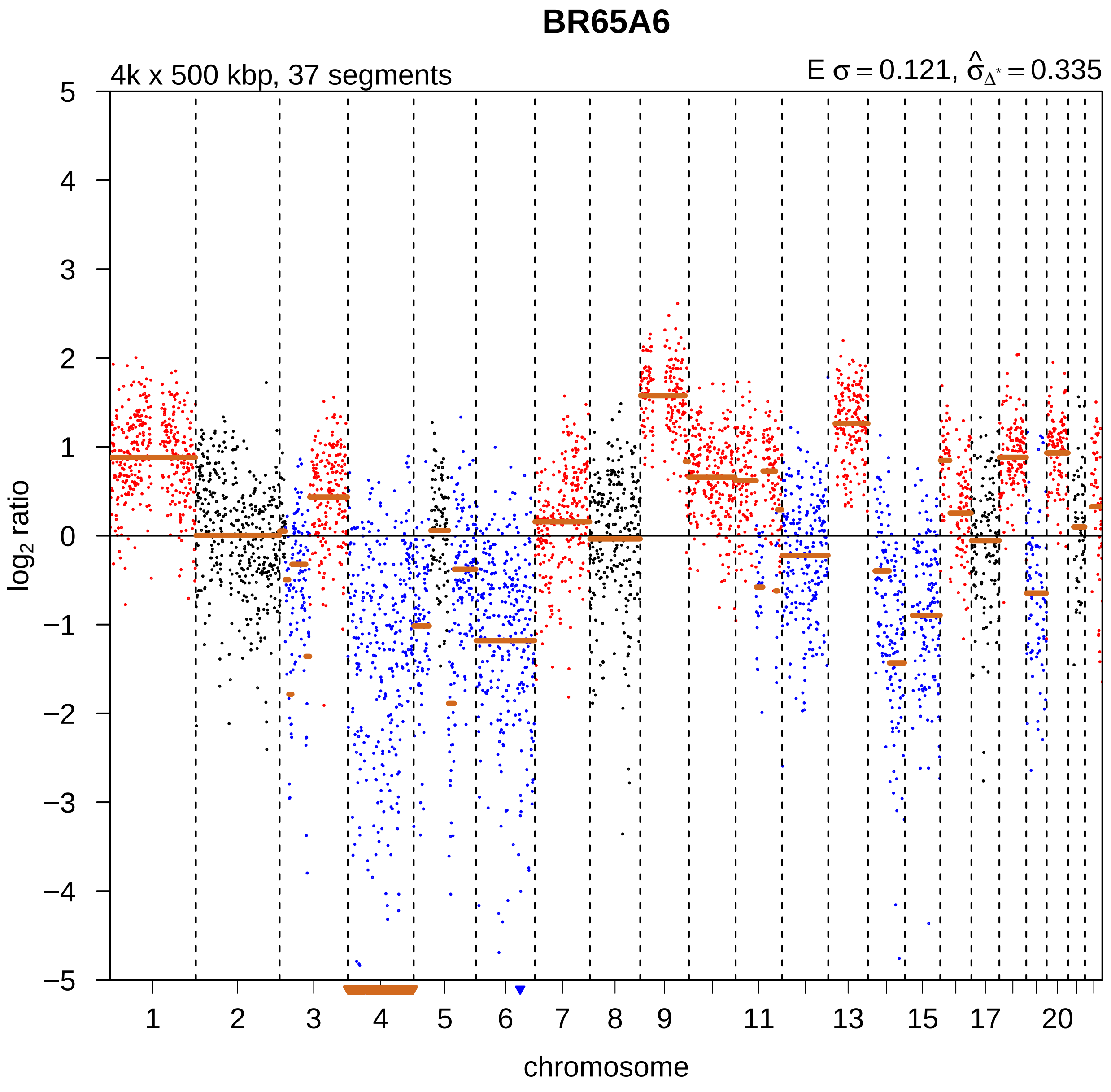

BR65A6
4k x 500 kbp, 37 segments
E σ = 0.121, σ̂Δ* = 0.335
5
4
3
2
1
0
−1
−2
−3
−4
−5
log2 ratio
1
2
3
4
5
6
7
8
9
11
13
15
17
20
chromosome

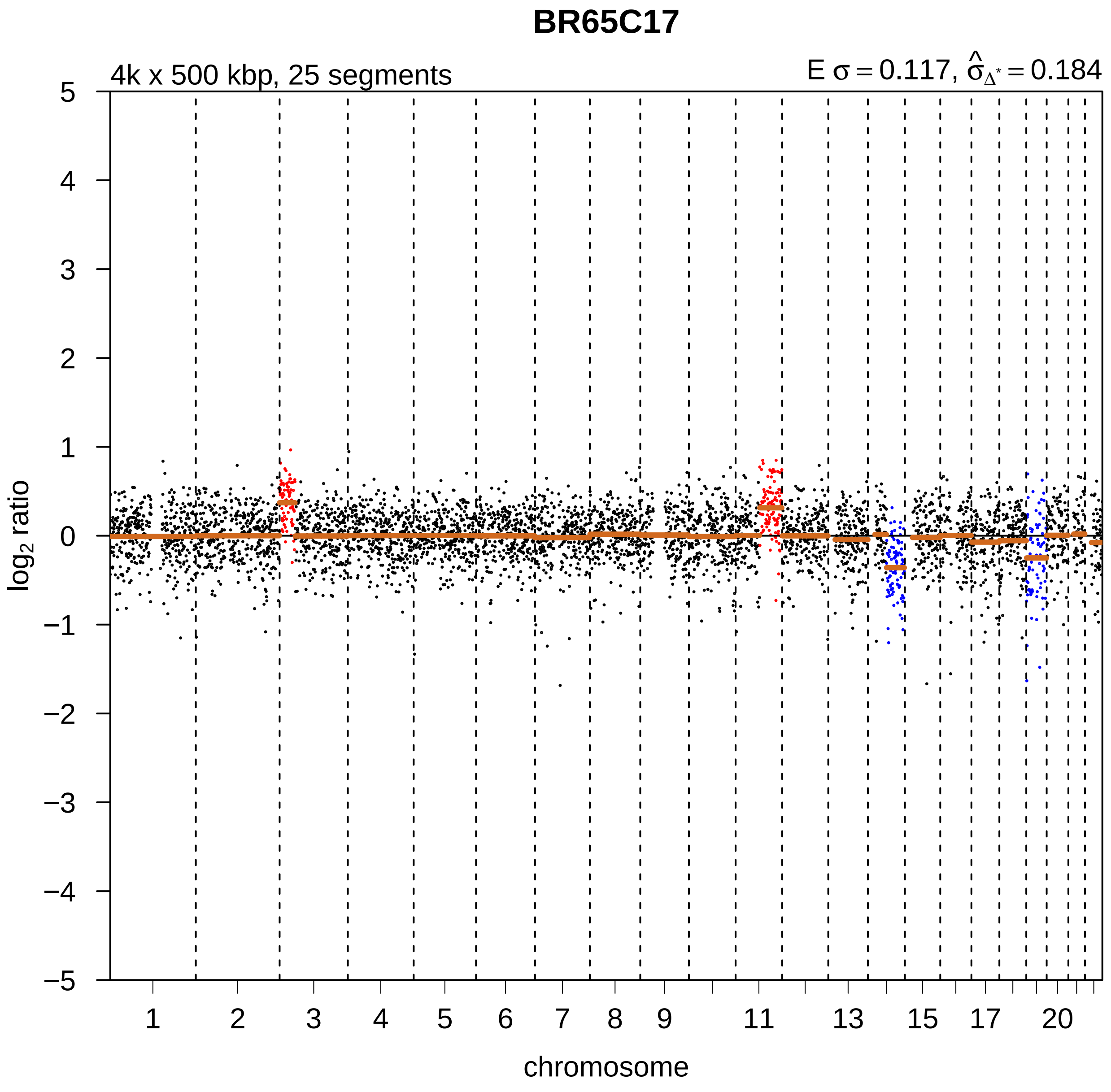

BR65C17
4k x 500 kbp, 25 segments
E σ = 0.117, σ̂_Δ* = 0.184
log2 ratio
5
4
3
2
1
0
−1
−2
−3
−4
−5
1
2
3
4
5
6
7
8
9
11
13
15
17
20
chromosome

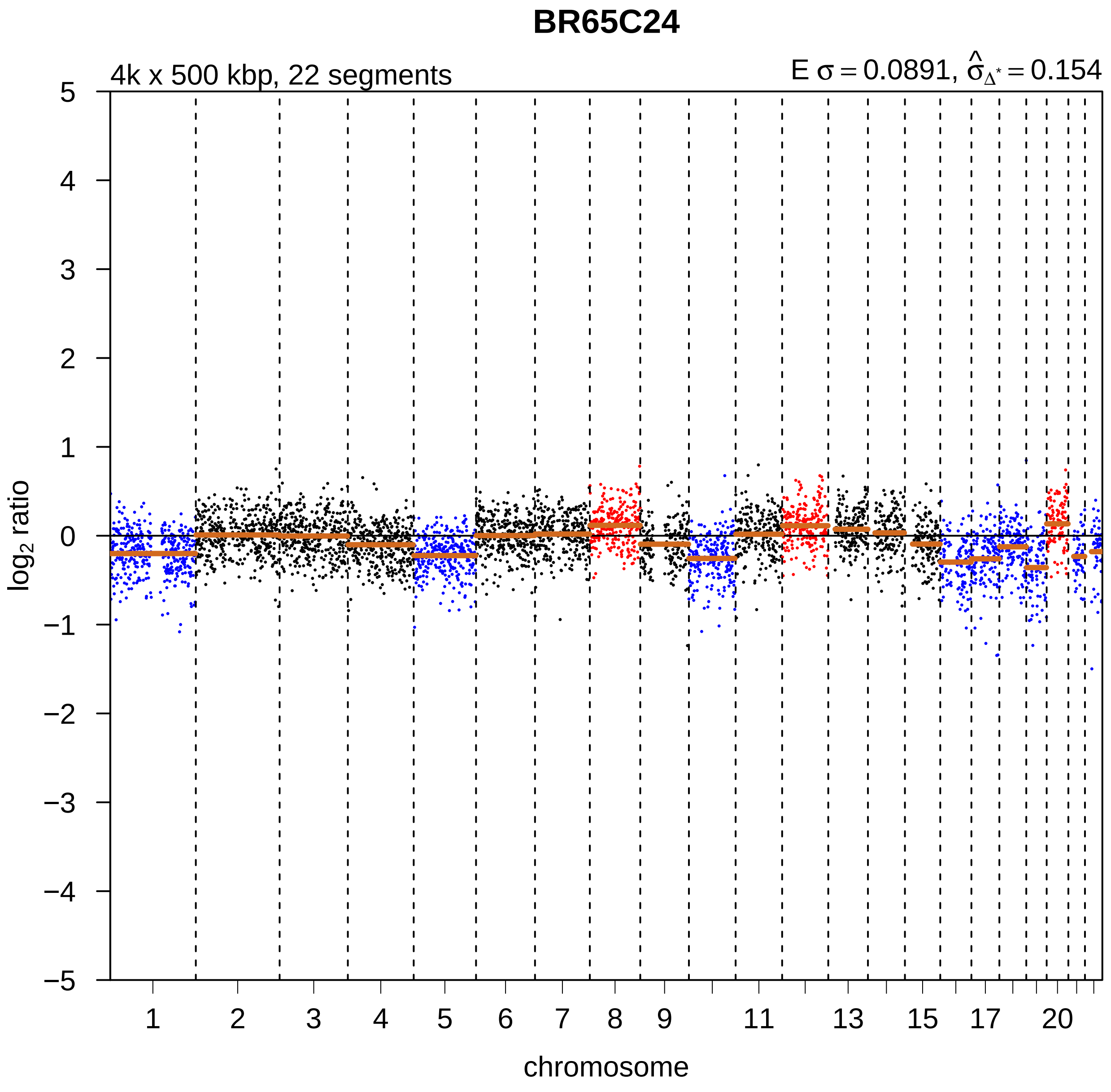
BR65C24
4k x 500 kbp, 22 segments
E σ = 0.0891, σ̂_Δ* = 0.154
log2 ratio
5
4
3
2
1
0
−1
−2
−3
−4
−5
1
2
3
4
5
6
7
8
9
11
13
15
17
20
chromosome

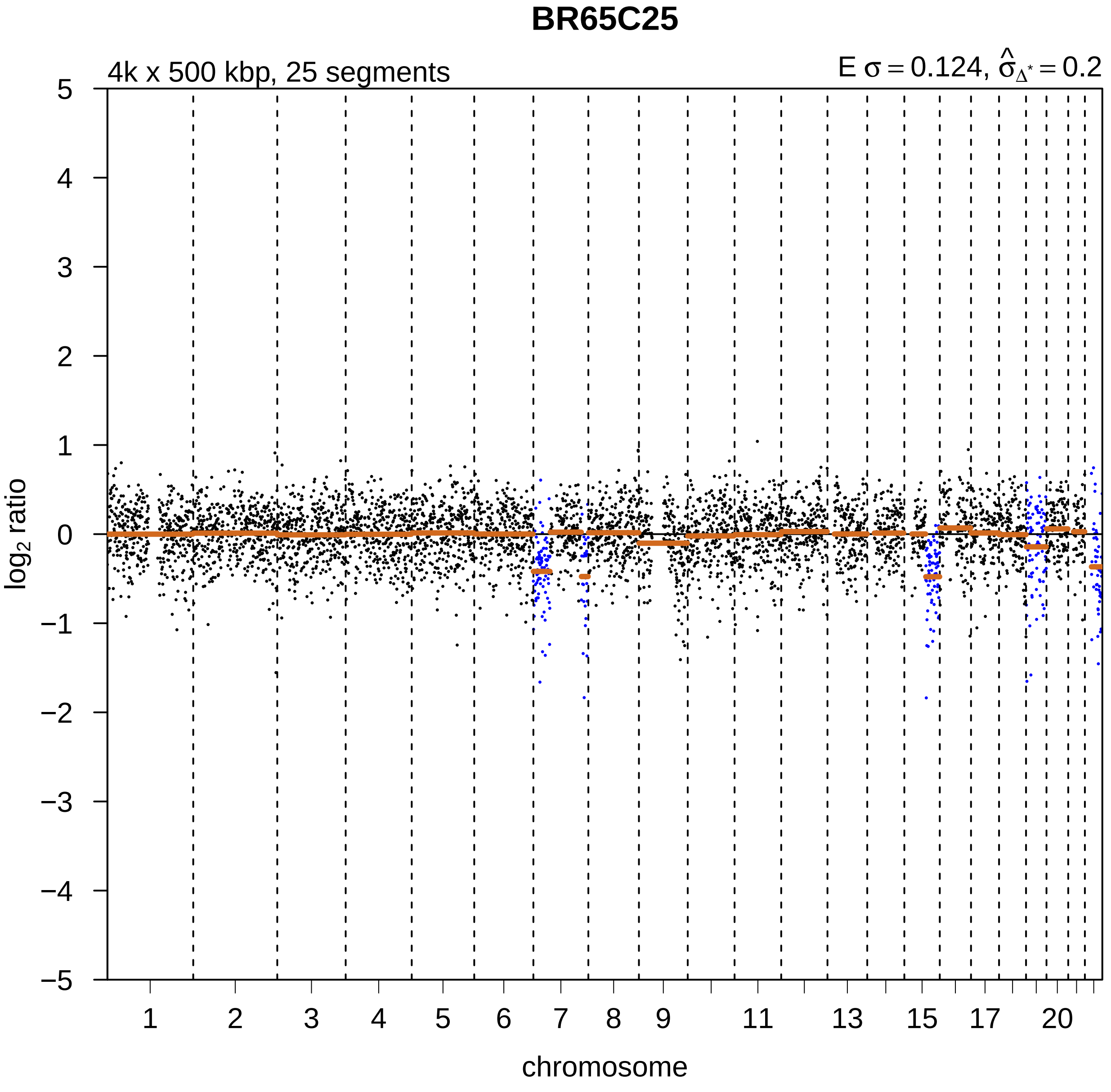
BR65C25
4k x 500 kbp, 25 segments
E σ = 0.124, σ̂Δ* = 0.2
log2 ratio
chromosome
5
4
3
2
1
0
−1
−2
−3
−4
−5
1
2
3
4
5
6
7
8
9
11
13
15
17
20

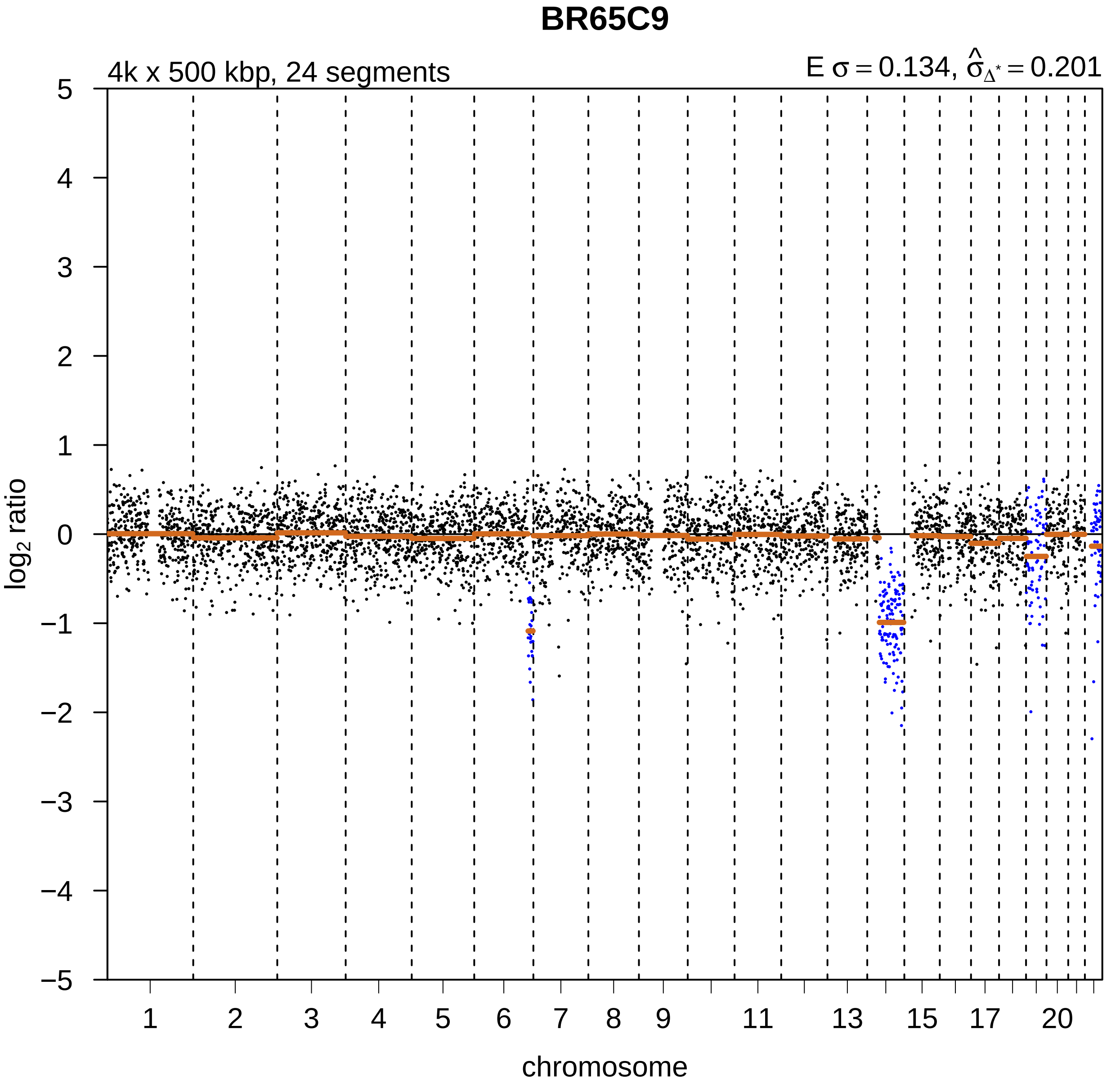
BR65C9
4k x 500 kbp, 24 segments
E σ = 0.134, $\hat{\sigma}_{\Delta^*}$ = 0.201
log2 ratio
chromosome
5
4
3
2
1
0
−1
−2
−3
−4
−5
1
2
3
4
5
6
7
8
9
11
13
15
17
20

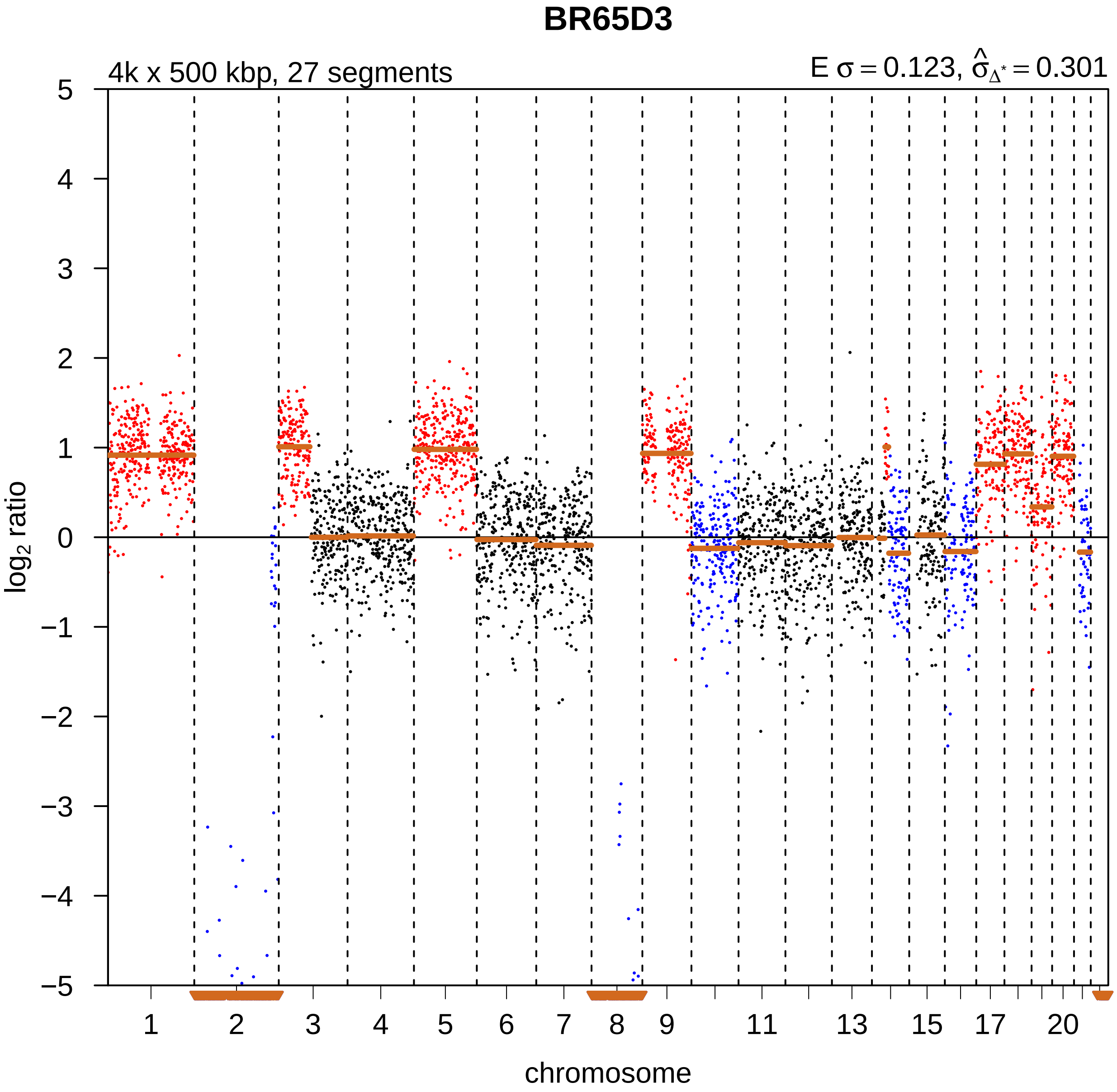

BR65D3
4k x 500 kbp, 27 segments
E σ = 0.123, $\hat{\sigma}_{\Delta^*}$ = 0.301
5
4
3
2
1
0
−1
−2
−3
−4
−5
log2 ratio
1
2
3
4
5
6
7
8
9
11
13
15
17
20
chromosome

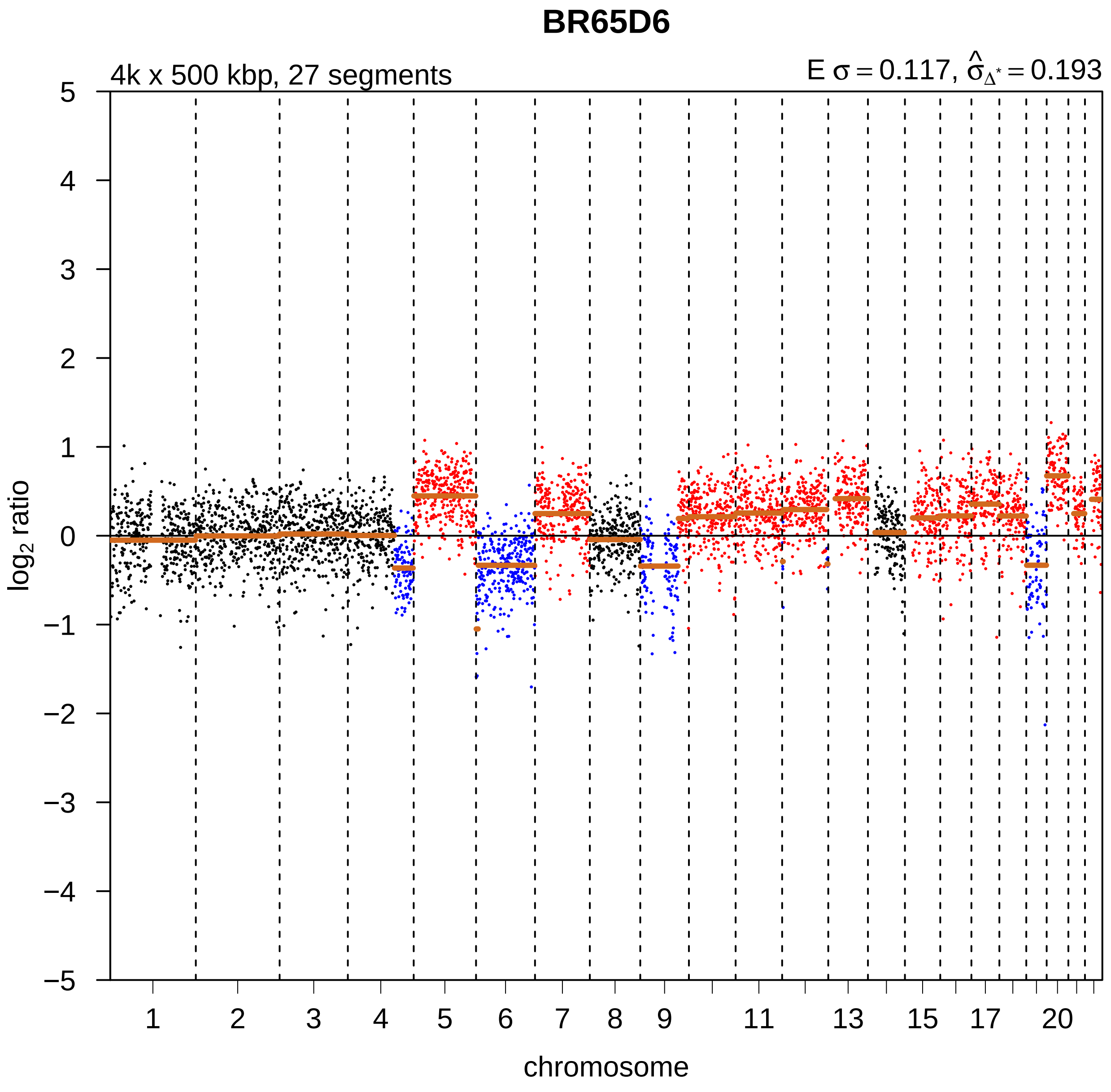
BR65D6
4k x 500 kbp, 27 segments
E σ = 0.117, σ̂Δ* = 0.193
log2 ratio
5
4
3
2
1
0
−1
−2
−3
−4
−5
1
2
3
4
5
6
7
8
9
11
13
15
17
20
chromosome

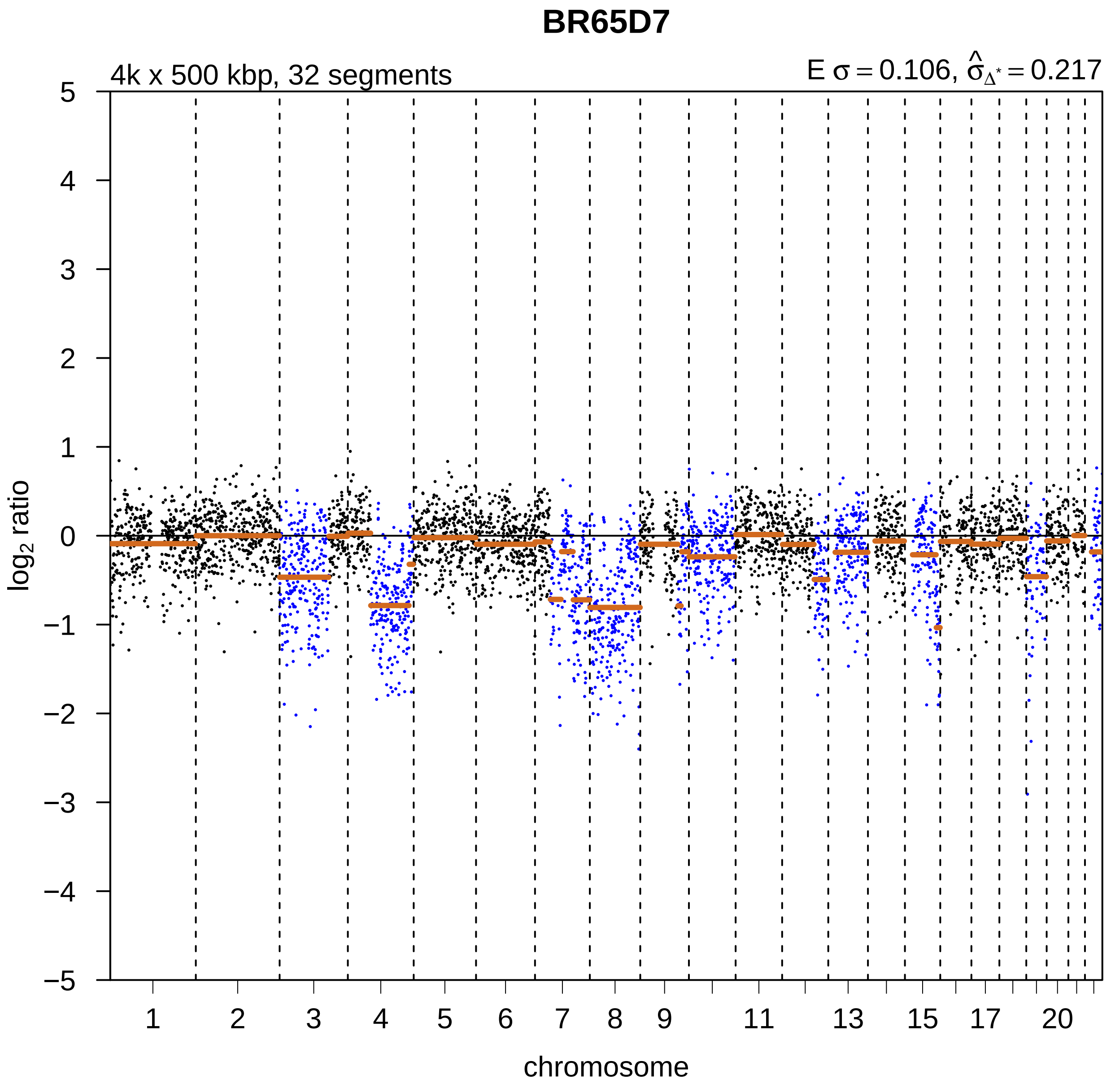

BR65D7
4k x 500 kbp, 32 segments
E σ = 0.106, $\hat{\sigma}_{\Delta^*}$ = 0.217
log2 ratio
5
4
3
2
1
0
−1
−2
−3
−4
−5
1
2
3
4
5
6
7
8
9
11
13
15
17
20
chromosome

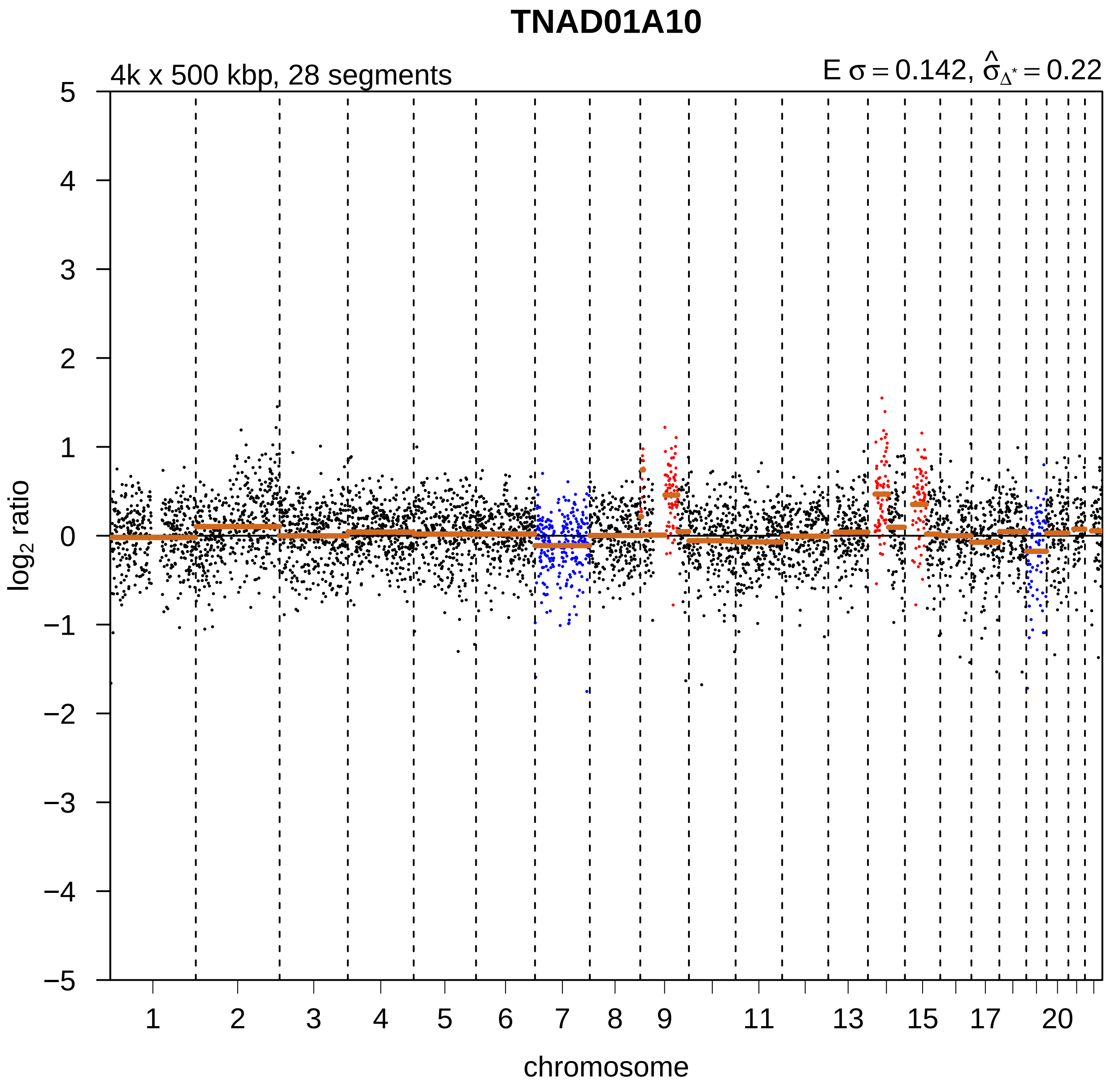

TNAD01A10
4k x 500 kbp, 28 segments
E σ = 0.142, $\hat{\sigma}_{\Delta^*}$ = 0.22
log2 ratio
5
4
3
2
1
0
−1
−2
−3
−4
−5
1
2
3
4
5
6
7
8
9
11
13
15
17
20
chromosome

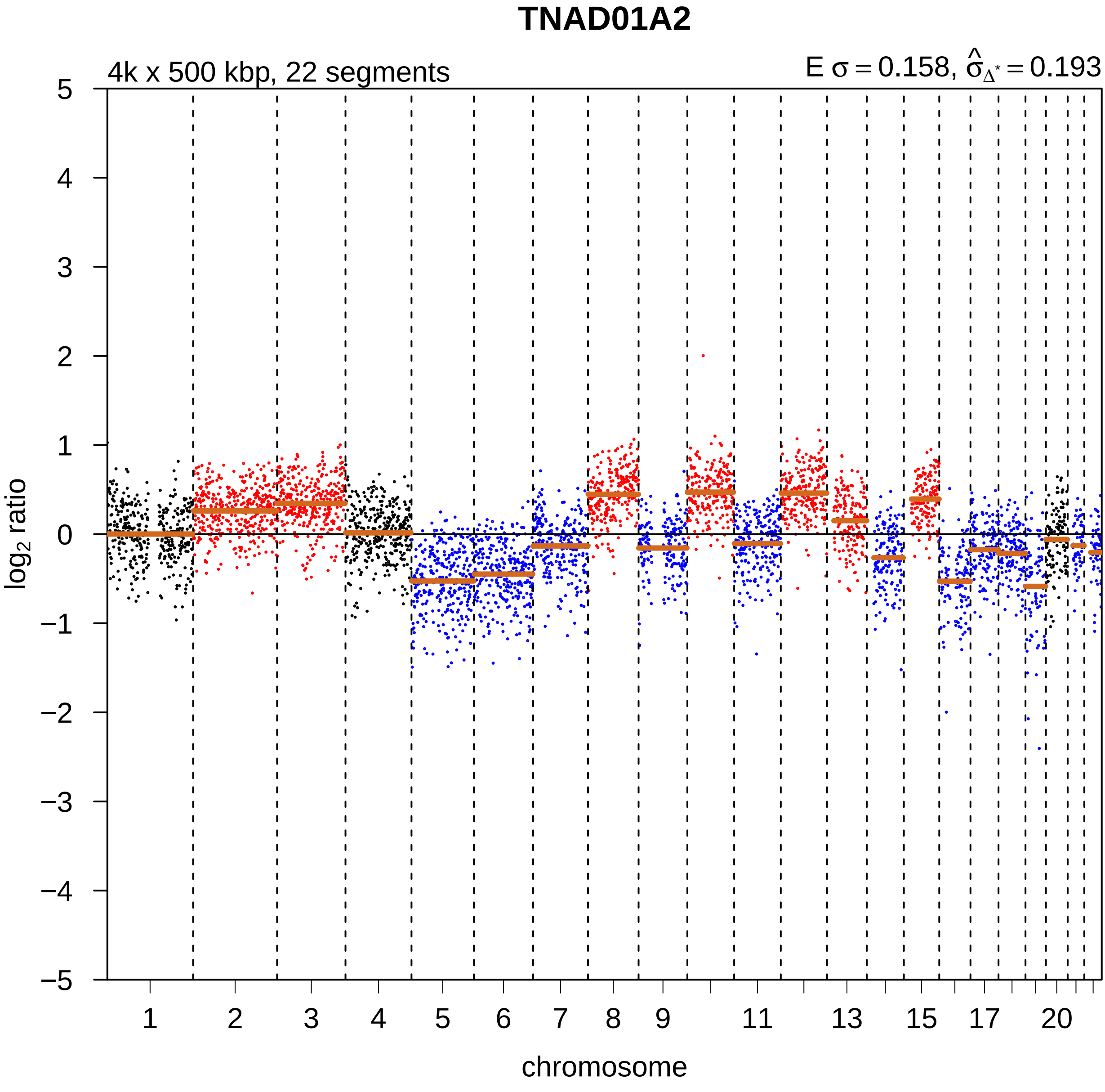

TNAD01A2
4k x 500 kbp, 22 segments
$E\ \sigma = 0.158,\ \hat{\sigma}_{\Delta^*} = 0.193$
log$_2$ ratio
5
4
3
2
1
0
−1
−2
−3
−4
−5
1
2
3
4
5
6
7
8
9
11
13
15
17
20
chromosome

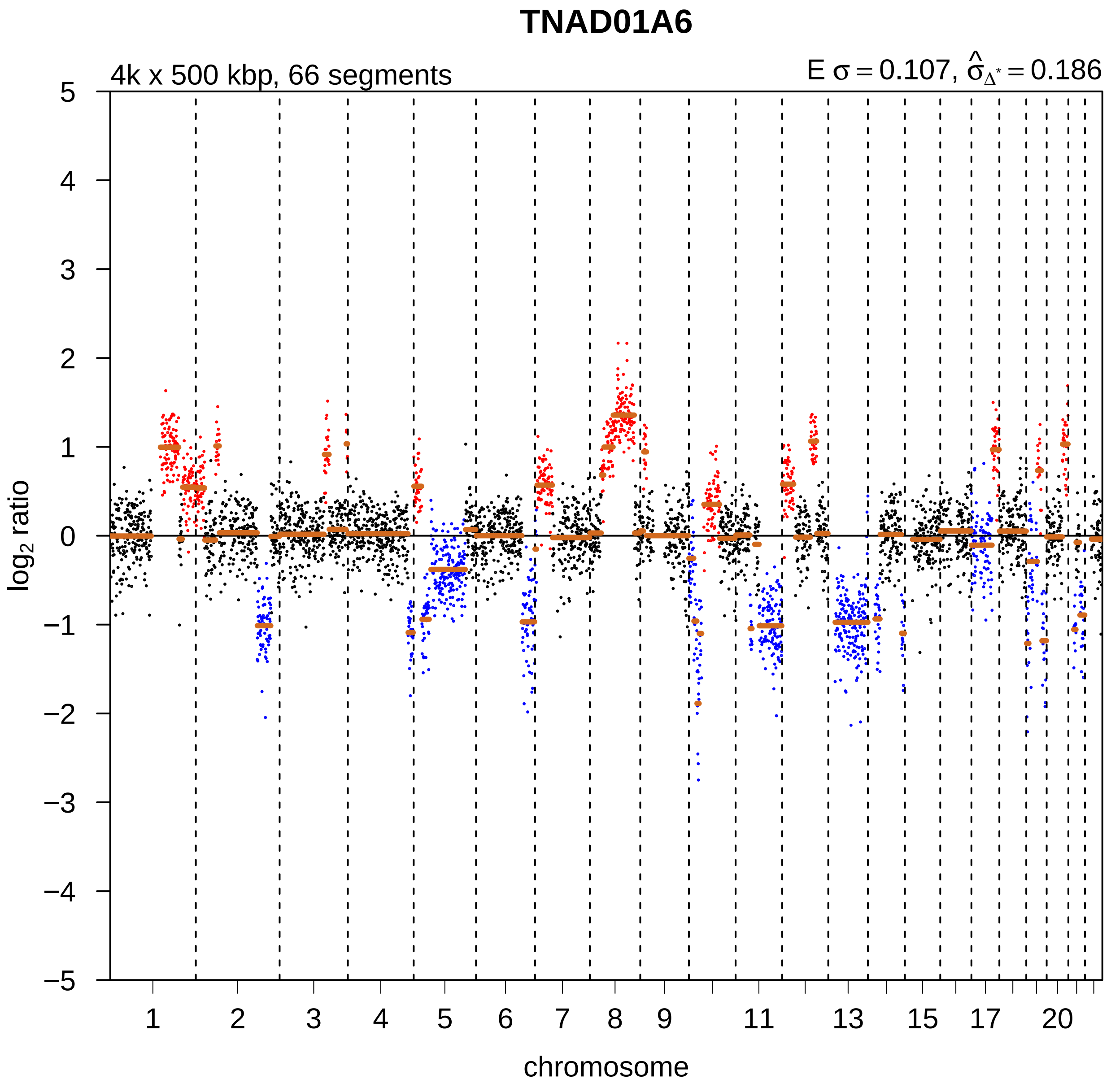
TNAD01A6
4k x 500 kbp, 66 segments
E σ = 0.107, $\hat{\sigma}_{\Delta^*}$ = 0.186
5
4
3
2
1
0
−1
−2
−3
−4
−5
log2 ratio
1
2
3
4
5
6
7
8
9
11
13
15
17
20
chromosome

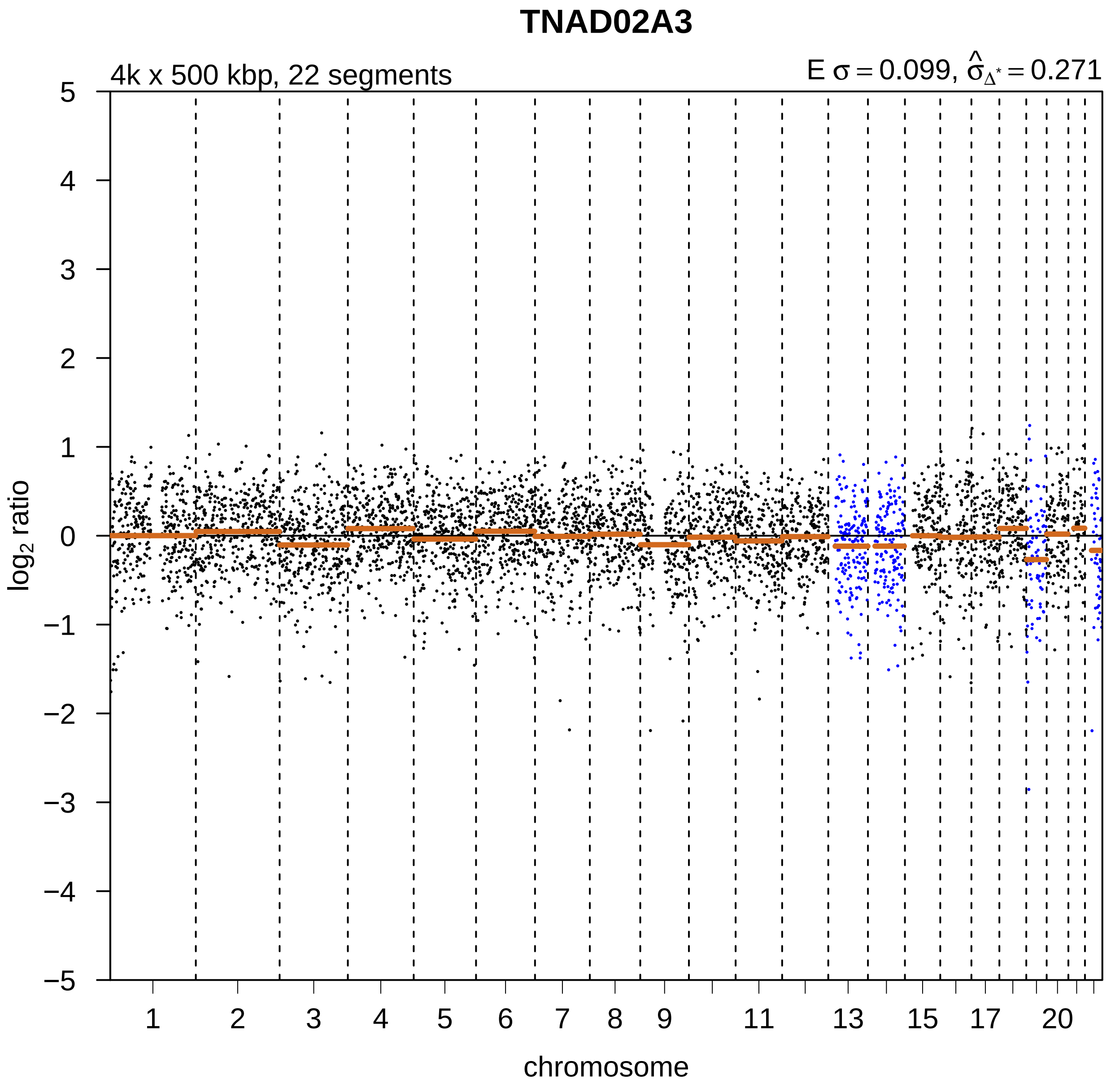
TNAD02A3
4k x 500 kbp, 22 segments
E σ = 0.099, σ̂Δ* = 0.271
log2 ratio
chromosome
5
4
3
2
1
0
−1
−2
−3
−4
−5
1
2
3
4
5
6
7
8
9
11
13
15
17
20

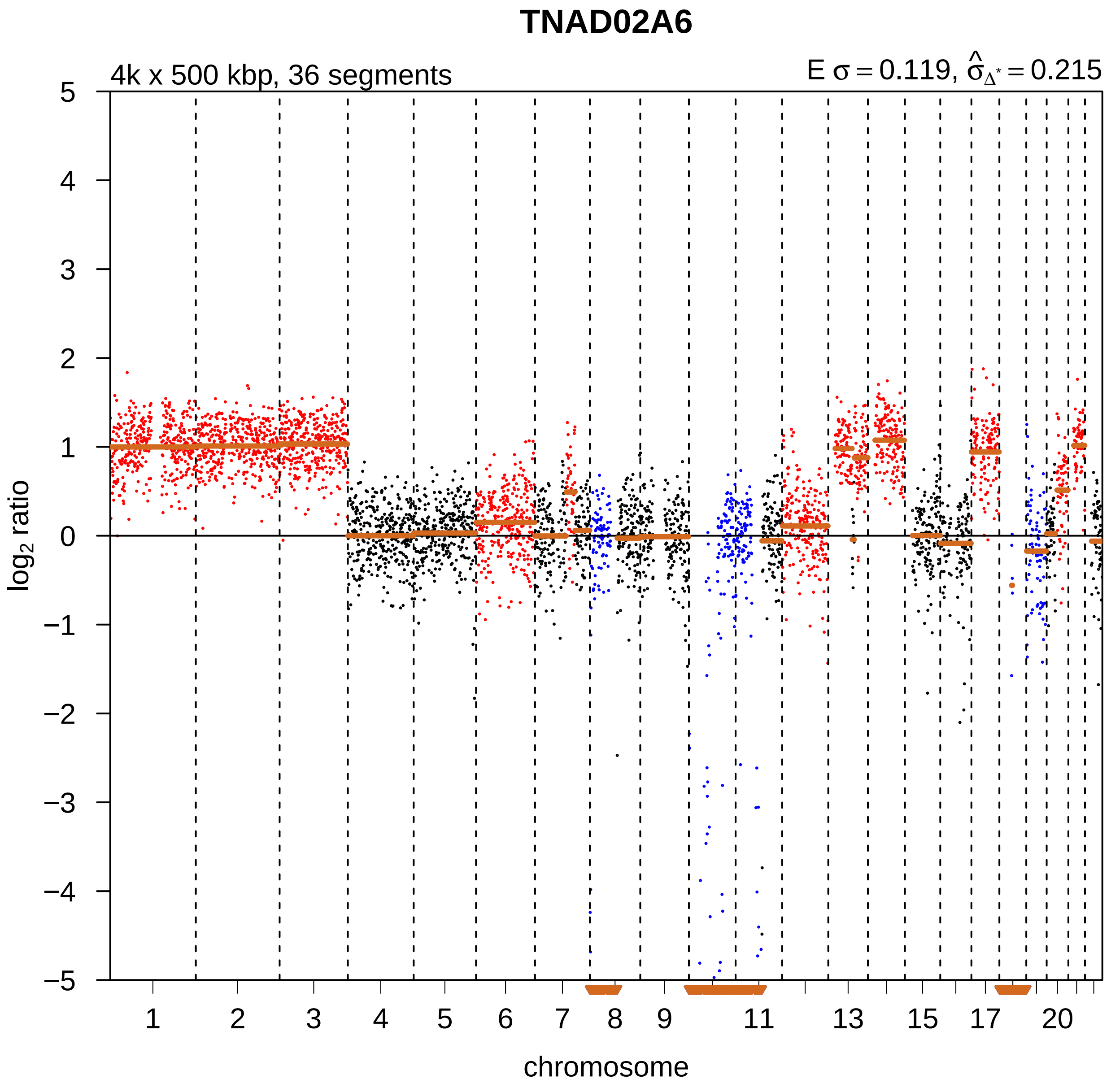

TNAD02A6
4k x 500 kbp, 36 segments
E σ = 0.119, $\hat{\sigma}_{\Delta^*}$ = 0.215
log2 ratio
5
4
3
2
1
0
−1
−2
−3
−4
−5
1 2 3 4 5 6 7 8 9 11 13 15 17 20
chromosome

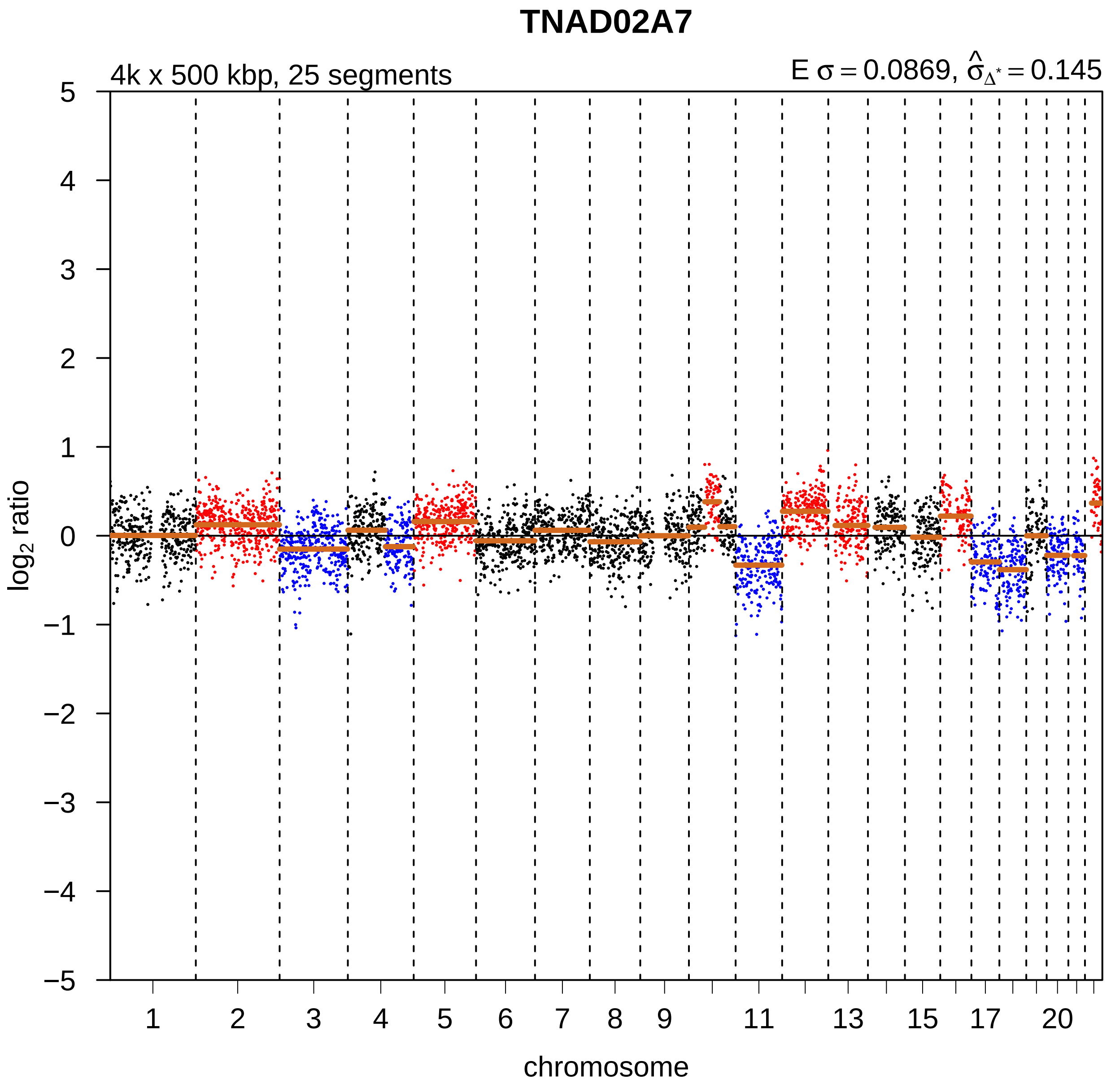
TNAD02A7
4k x 500 kbp, 25 segments
E σ = 0.0869, σ̂Δ* = 0.145
log2 ratio
5
4
3
2
1
0
−1
−2
−3
−4
−5
1
2
3
4
5
6
7
8
9
11
13
15
17
20
chromosome

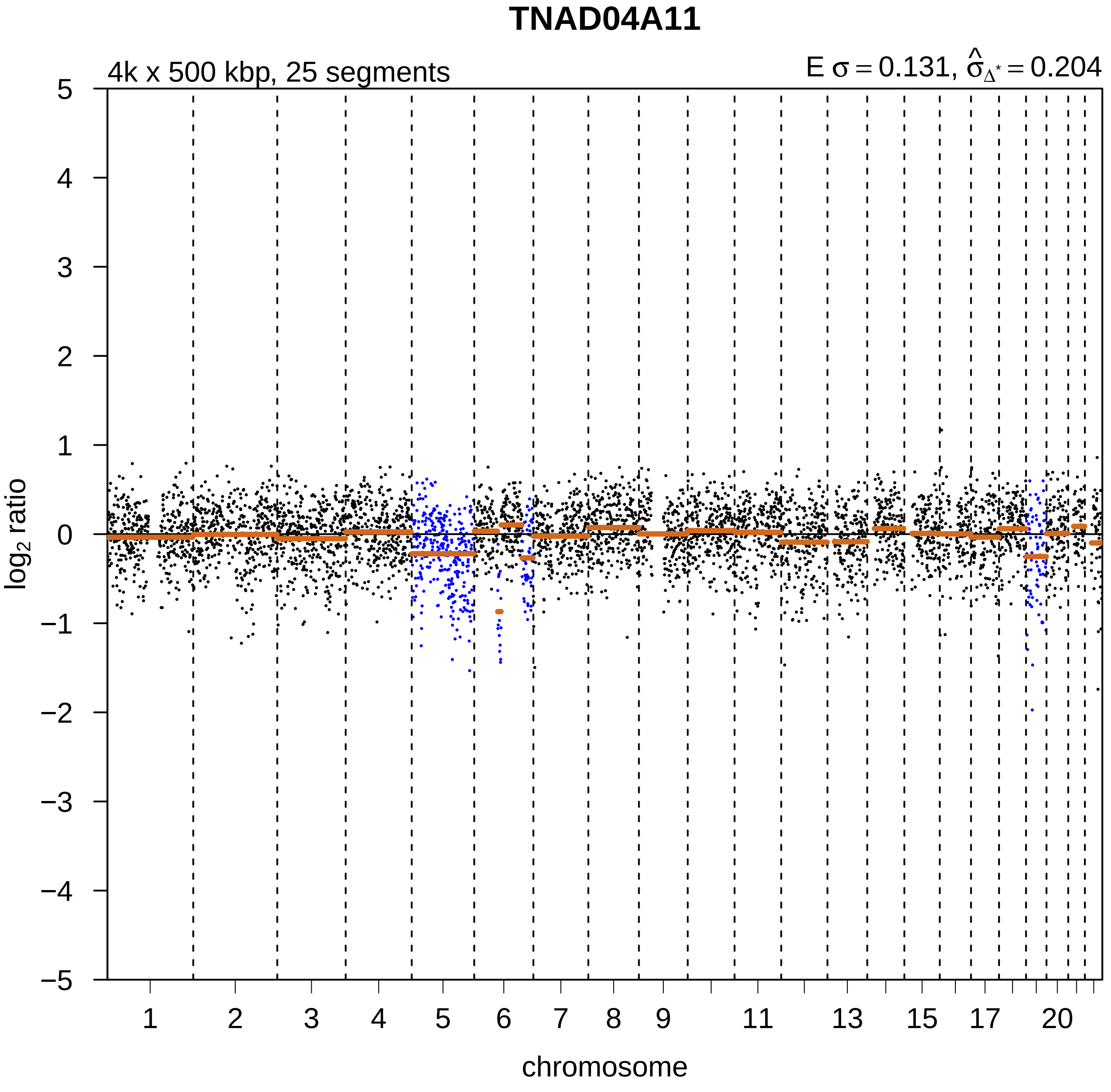

TNAD04A11
4k x 500 kbp, 25 segments
E σ = 0.131, σ̂_Δ* = 0.204
5
4
3
2
1
0
−1
−2
−3
−4
−5
log2 ratio
1
2
3
4
5
6
7
8
9
11
13
15
17
20
chromosome

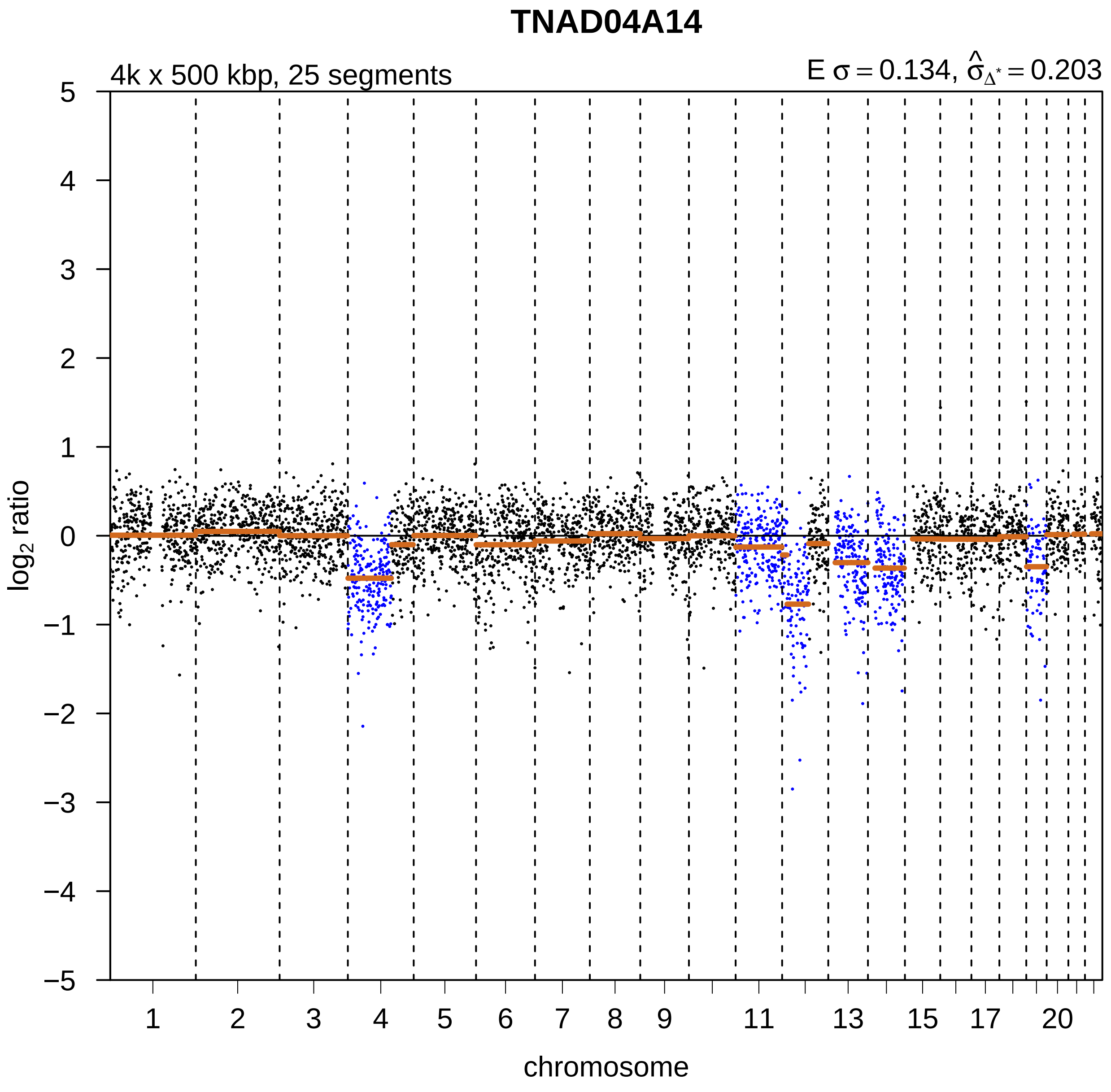

TNAD04A14
4k x 500 kbp, 25 segments
E σ = 0.134, $\hat{\sigma}_{\Delta^*}$ = 0.203
log2 ratio
5
4
3
2
1
0
−1
−2
−3
−4
−5
1
2
3
4
5
6
7
8
9
11
13
15
17
20
chromosome

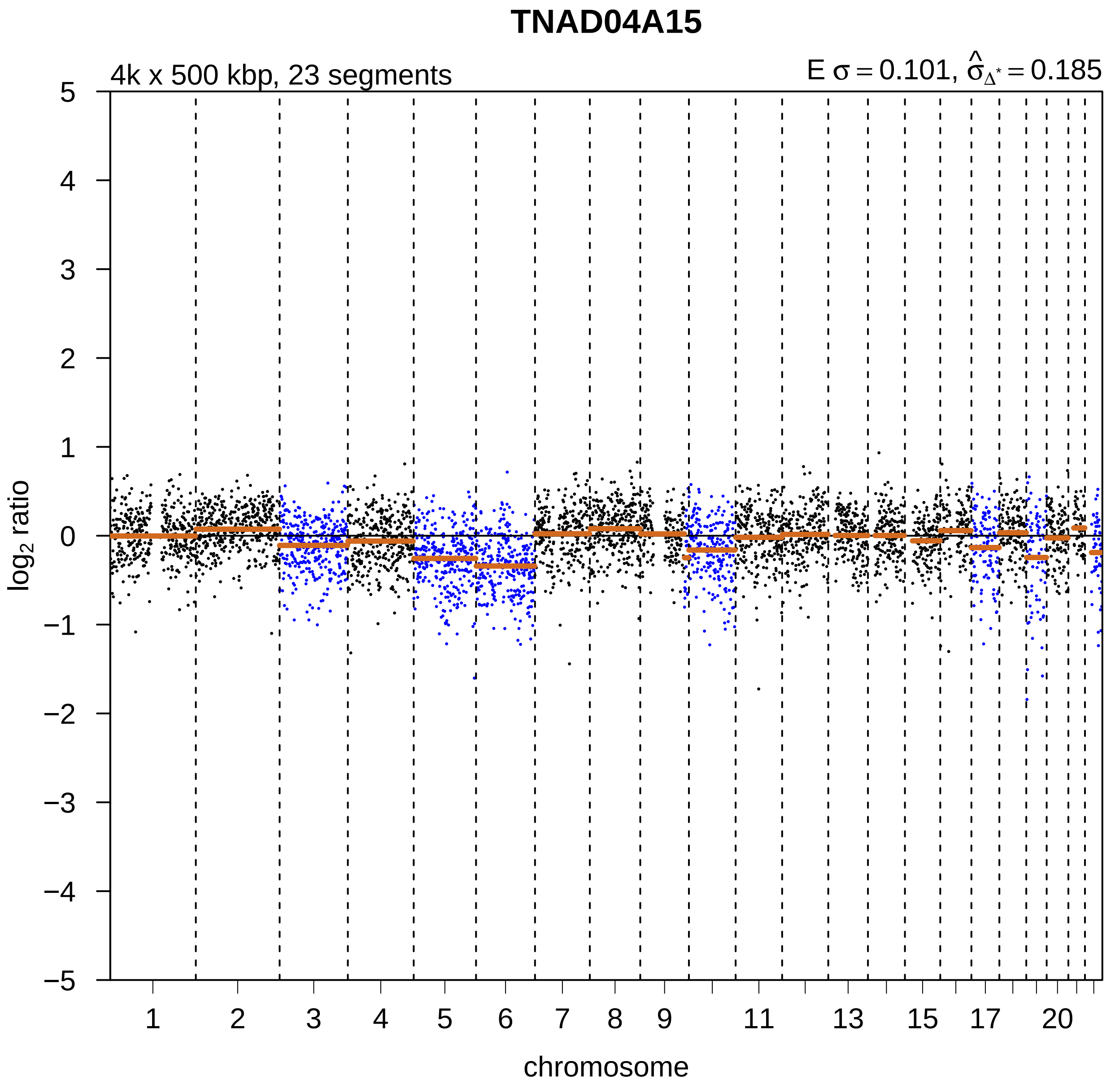
TNAD04A15
4k x 500 kbp, 23 segments
E σ = 0.101, $\hat{\sigma}_{\Delta^*}$ = 0.185
5
4
3
2
1
0
−1
−2
−3
−4
−5
log2 ratio
1
2
3
4
5
6
7
8
9
11
13
15
17
20
chromosome

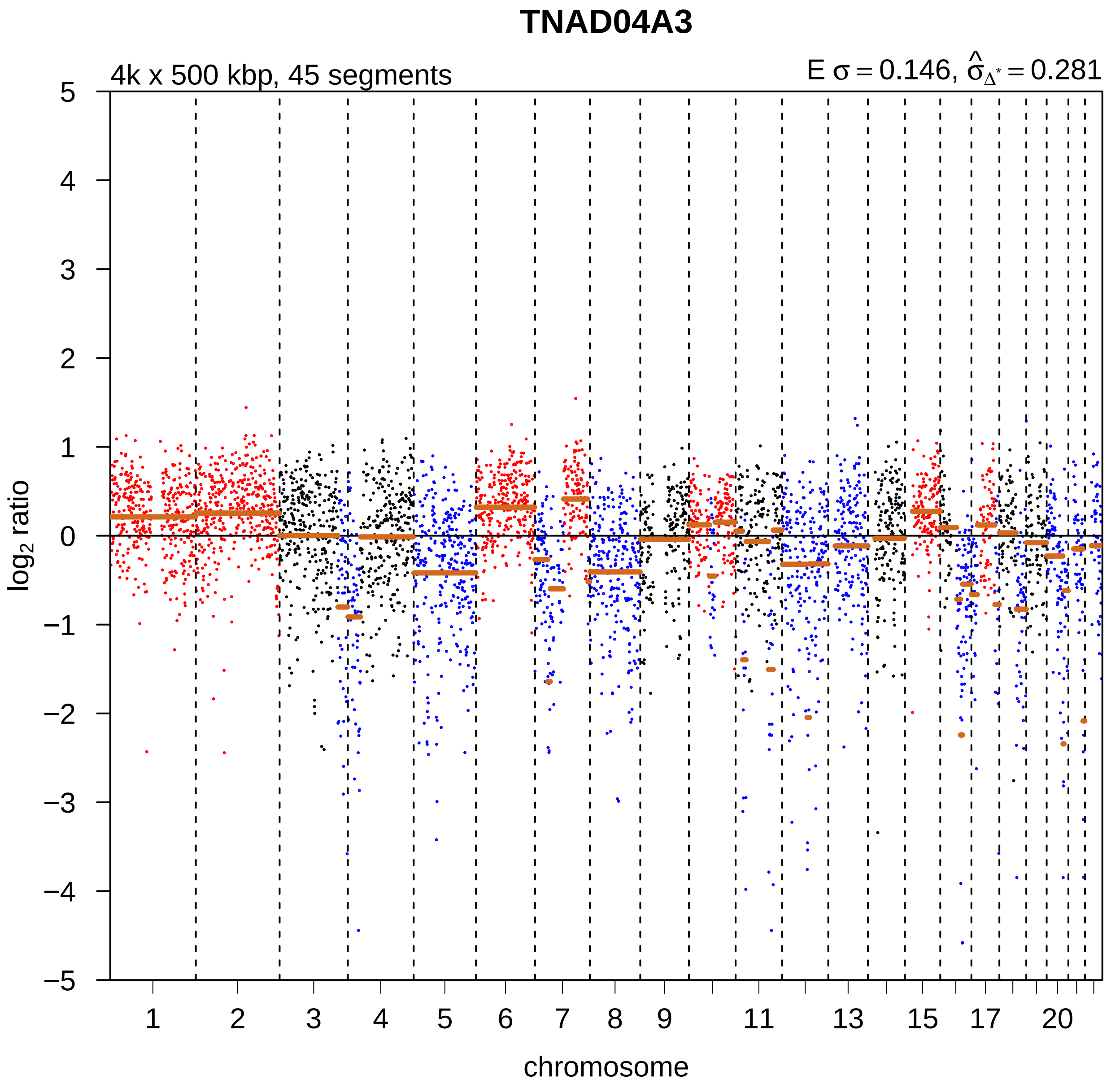

TNAD04A3
4k x 500 kbp, 45 segments
E σ = 0.146, $\hat{\sigma}_{\Delta^*}$ = 0.281
5
4
3
2
1
0
−1
−2
−3
−4
−5
$\log_2$ ratio
1
2
3
4
5
6
7
8
9
11
13
15
17
20
chromosome

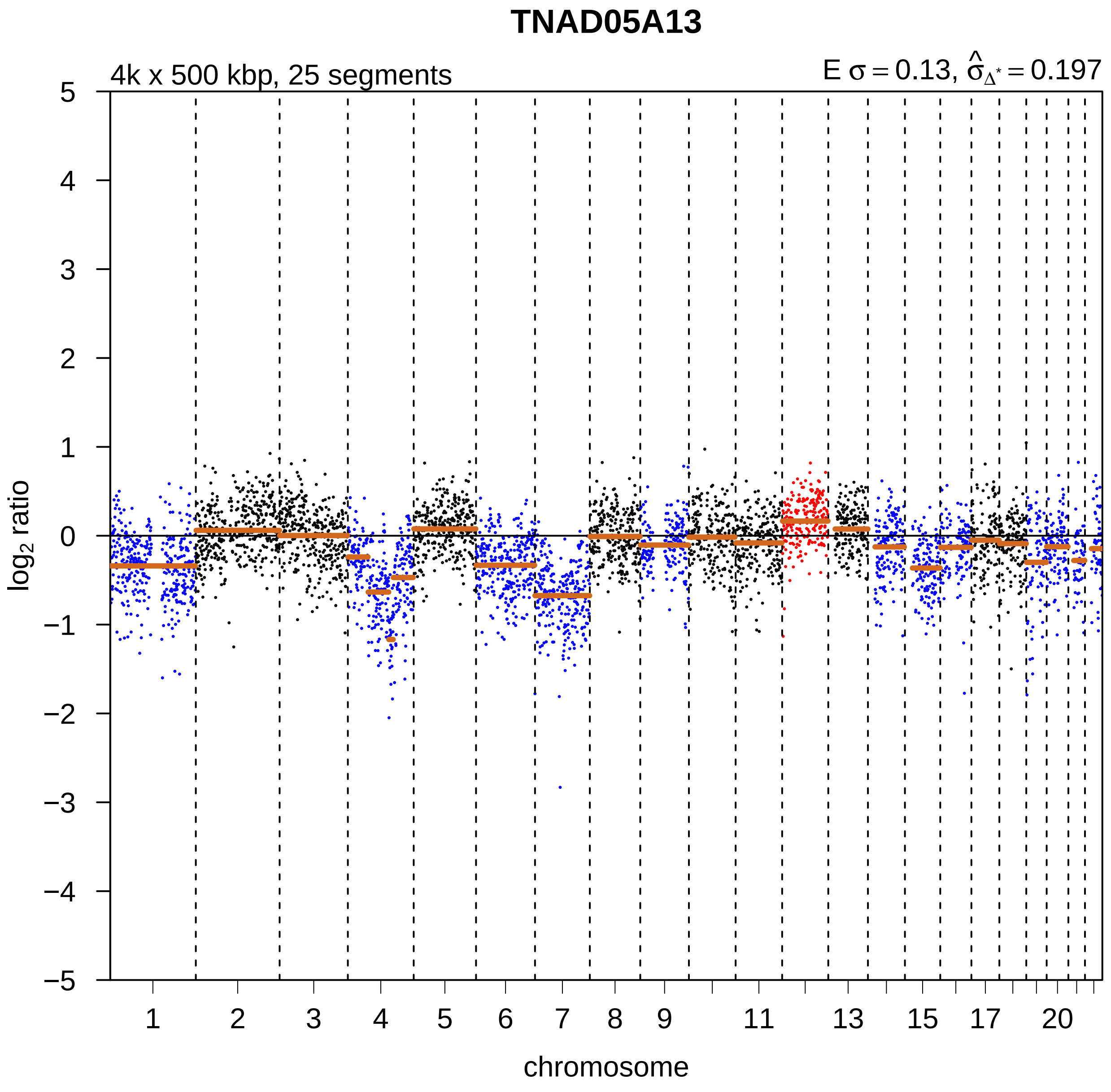
TNAD05A13
4k x 500 kbp, 25 segments
E σ = 0.13, σ̂Δ* = 0.197
log2 ratio
chromosome
5
4
3
2
1
0
−1
−2
−3
−4
−5
1
2
3
4
5
6
7
8
9
11
13
15
17
20

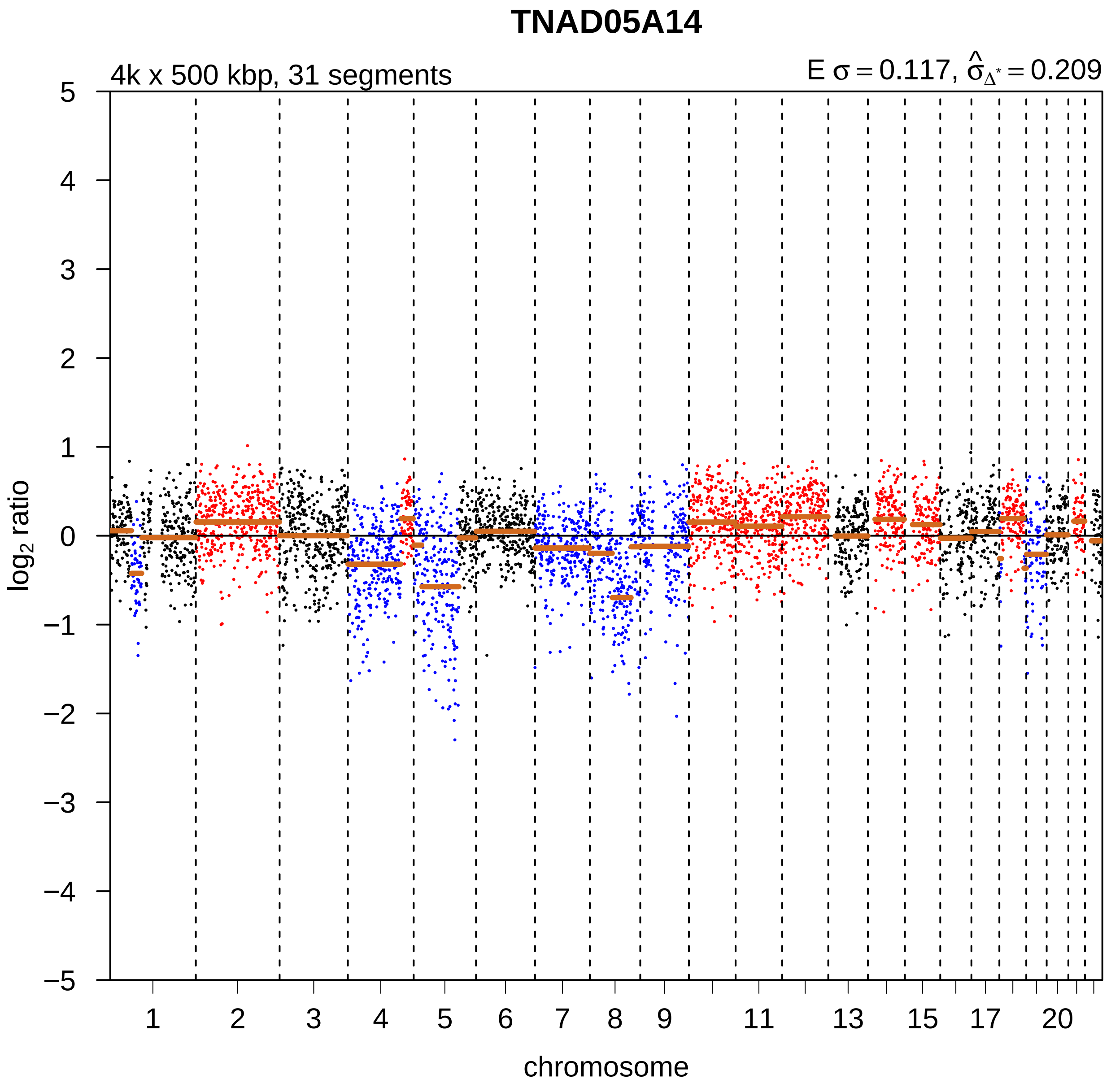
TNAD05A14
4k x 500 kbp, 31 segments
E σ = 0.117, $\hat{\sigma}_{\Delta^*}$ = 0.209
log2 ratio
5
4
3
2
1
0
−1
−2
−3
−4
−5
1
2
3
4
5
6
7
8
9
11
13
15
17
20
chromosome

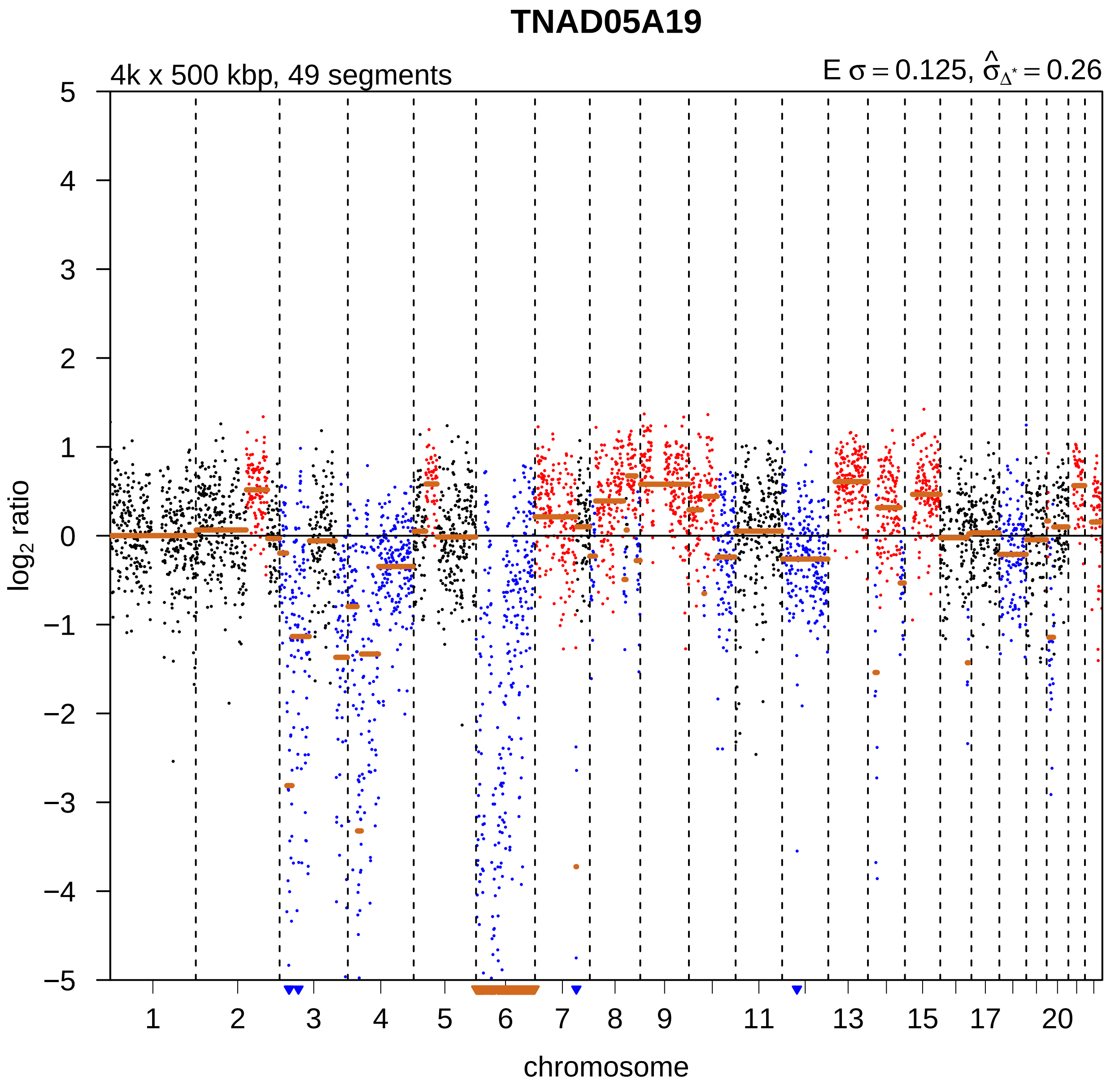
TNAD05A19
4k x 500 kbp, 49 segments
E σ = 0.125, $\hat{\sigma}_{\Delta^*}$ = 0.26
log2 ratio
5
4
3
2
1
0
−1
−2
−3
−4
−5
1
2
3
4
5
6
7
8
9
11
13
15
17
20
chromosome

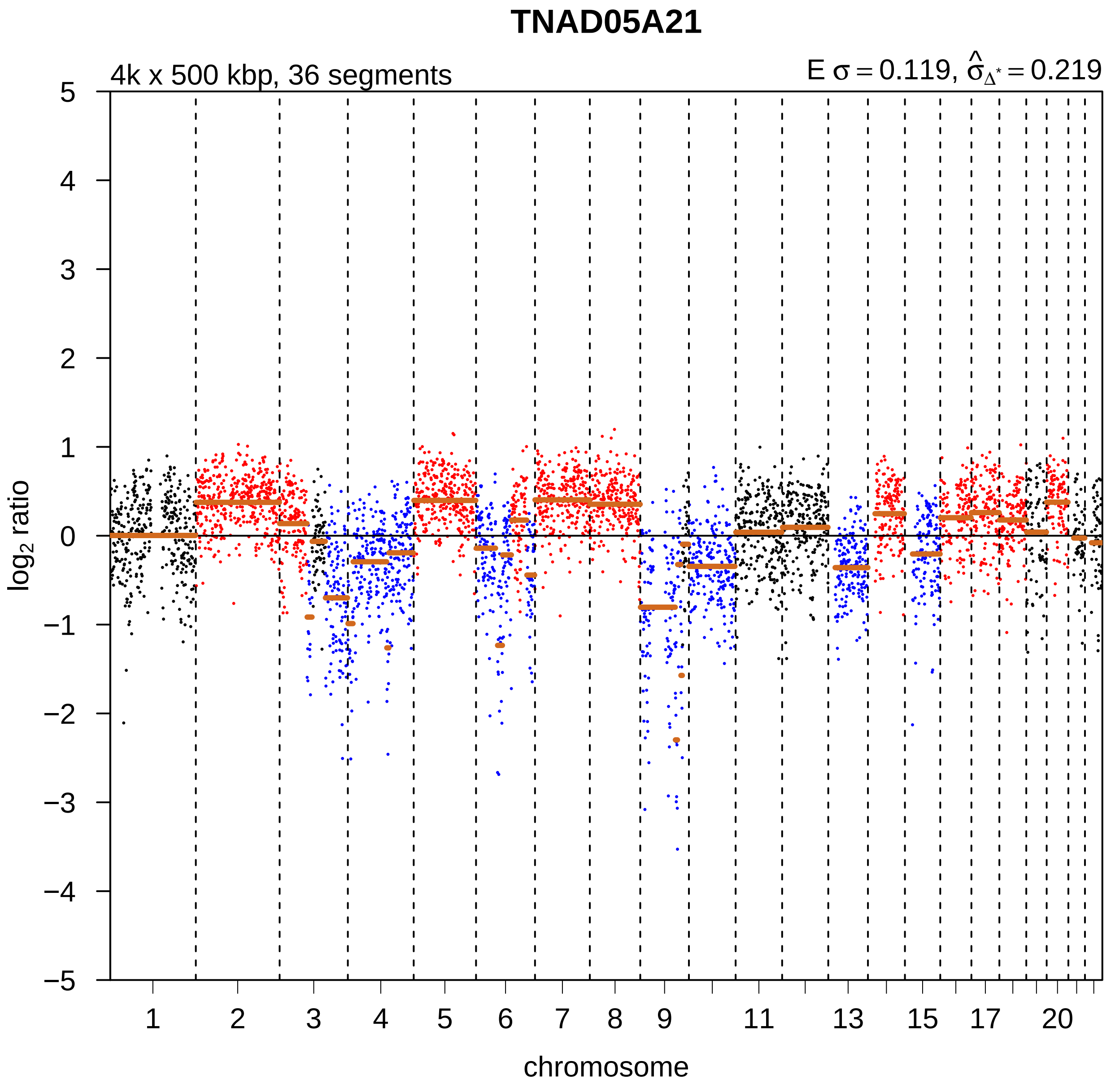
TNAD05A21
4k x 500 kbp, 36 segments
$E\,\sigma = 0.119,\ \hat{\sigma}_{\Delta^*} = 0.219$
log2 ratio
5
4
3
2
1
0
−1
−2
−3
−4
−5
1
2
3
4
5
6
7
8
9
11
13
15
17
20
chromosome

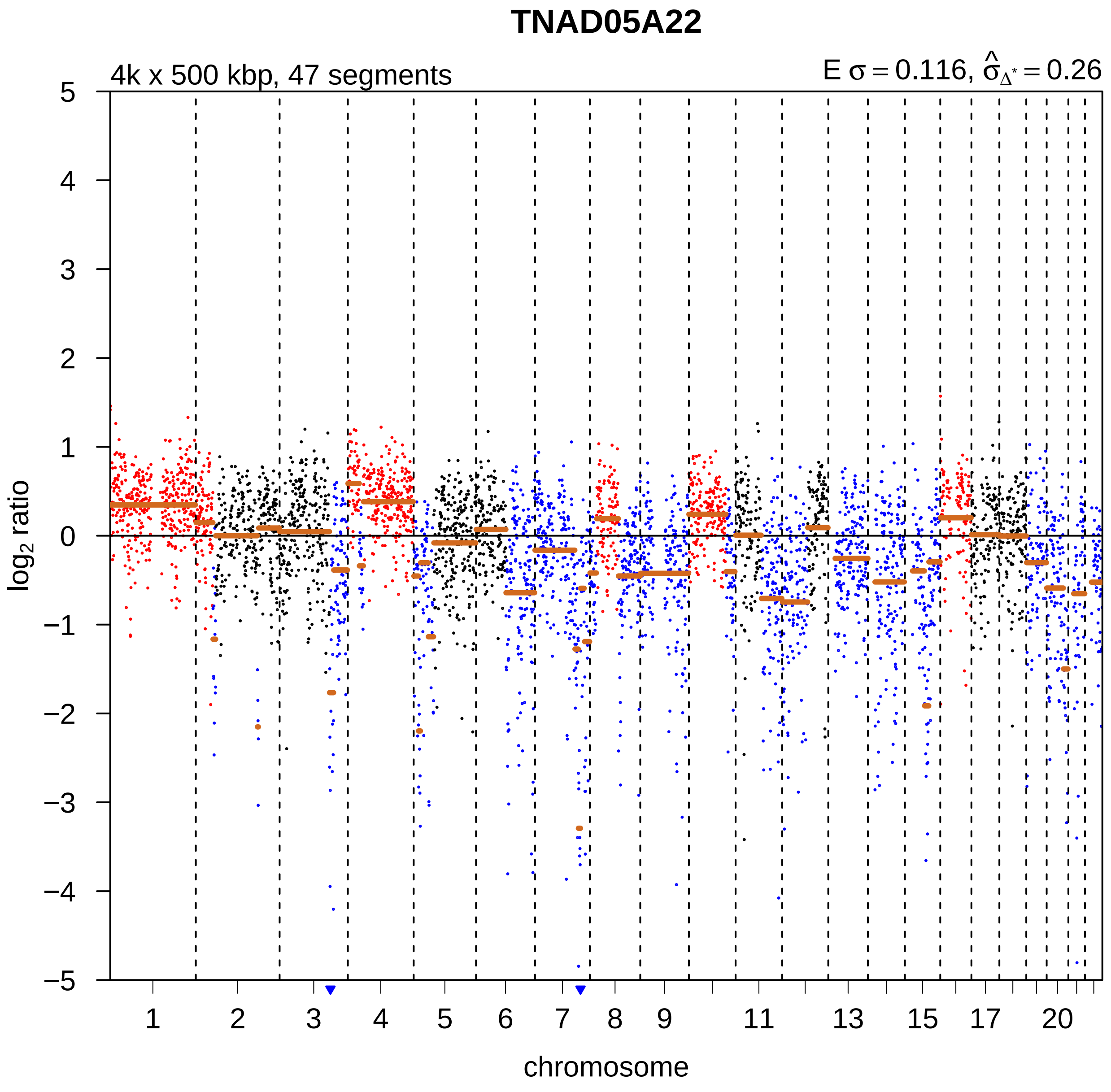

TNAD05A22
4k x 500 kbp, 47 segments
$E\,\sigma = 0.116,\ \hat{\sigma}_{\Delta^*} = 0.26$
log2 ratio
5
4
3
2
1
0
−1
−2
−3
−4
−5
1
2
3
4
5
6
7
8
9
11
13
15
17
20
chromosome

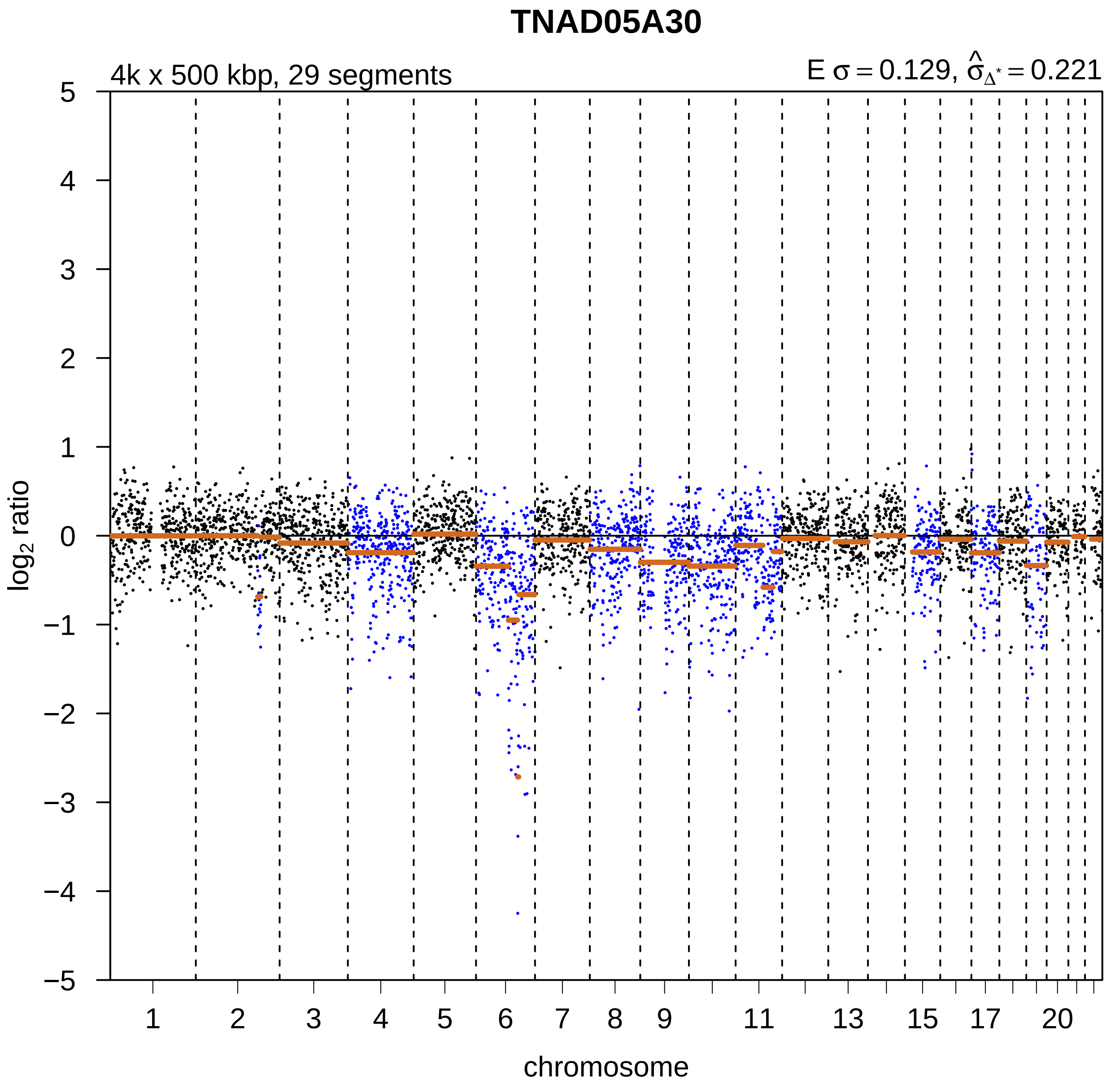
TNAD05A30
4k x 500 kbp, 29 segments
E σ = 0.129, σ̂_Δ* = 0.221
log2 ratio
5
4
3
2
1
0
−1
−2
−3
−4
−5
1
2
3
4
5
6
7
8
9
11
13
15
17
20
chromosome

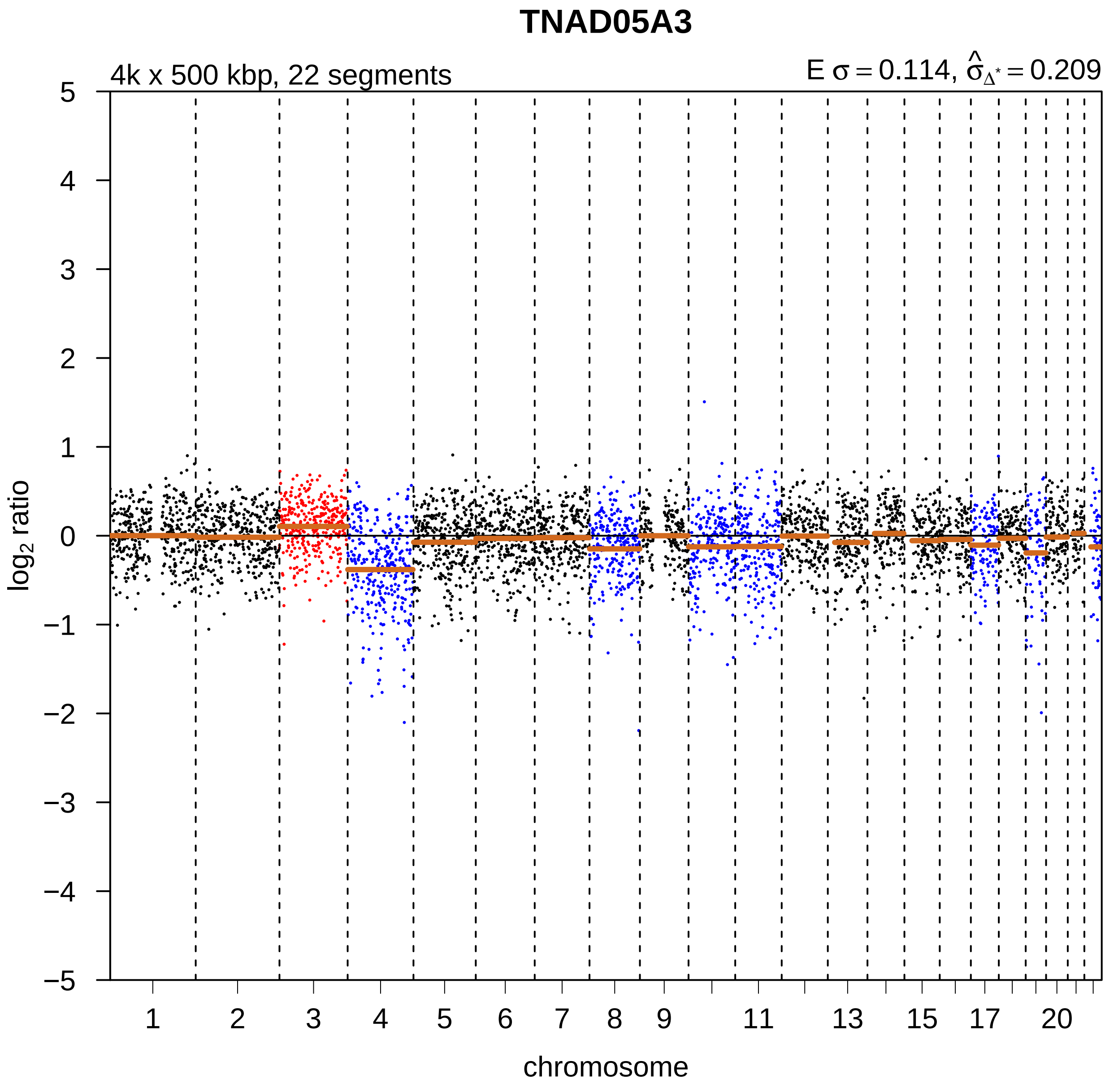
TNAD05A3
4k x 500 kbp, 22 segments
E σ = 0.114, $\hat{\sigma}_{\Delta^*}$ = 0.209
log2 ratio
5
4
3
2
1
0
−1
−2
−3
−4
−5
chromosome
1
2
3
4
5
6
7
8
9
11
13
15
17
20

TNAD05A8

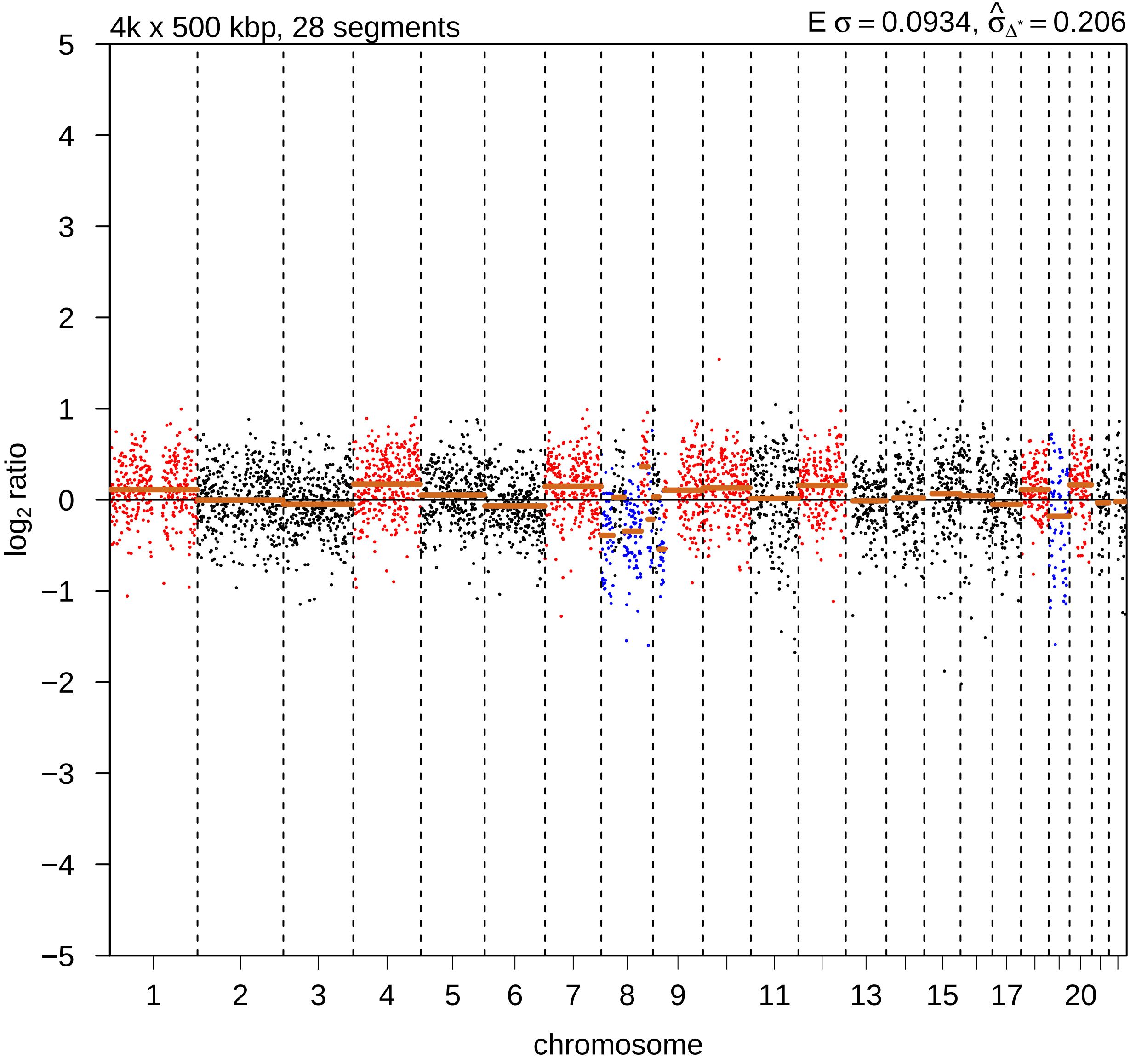

4k x 500 kbp, 28 segments
E σ = 0.0934, $\hat{\sigma}_{\Delta^*}$ = 0.206
5
4
3
2
1
0
−1
−2
−3
−4
−5
log2 ratio
1
2
3
4
5
6
7
8
9
11
13
15
17
20
chromosome
